# Periodontitis in Patients With Severe Obesity: From the Oral and Gut Microbiota Dysregulation to the Visceral Adipose Tissue Inflammatory and Metabolic Disorders

**DOI:** 10.1096/fj.202600054R

**Published:** 2026-04-27

**Authors:** Katy Thouvenot, Flavie Serrat, Victorine Lenclume, Eric Doussiet, Eugeni Belda, Janice Taïlé, Rohia Alili, Philippe Rondeau, Karine Clément, Olivier Meilhac, Nathalie Le Moullec, Marie‐Paule Gonthier

**Affiliations:** ^1^ INSERM, UMR 1188 Diabète athérothrombose Thérapies Réunion Océan Indien (DéTROI) Université de La Réunion Saint‐Pierre La Réunion France; ^2^ CHU de La Réunion Saint‐Pierre La Réunion France; ^3^ INSERM Centre d'Investigation Clinique (CIC) 1410 Saint‐Pierre La Réunion France; ^4^ INSERM, UMRS 1269 Nutrition and Obesities: Systemic Approaches (NutriOmique) Sorbonne Université Paris France; ^5^ Assistance Publique‐Hôpitaux de Paris, Département de Nutrition Hôpital Pitié‐Salpêtrière Paris France

**Keywords:** adipose tissue, inflammation, lipidemia, obesity, oral and gut microbiota, oxidative stress

## Abstract

During periodontitis, pathogenic oral bacteria like *Porphyromonas gingivalis* may exert systemic effects directly by translocating into the bloodstream and indirectly by deregulating the gut microbiota, aggravating obesity‐related complications. This study aimed to evaluate the links between the periodontal infection, the oral and gut microbiota composition, and the inflammatory and metabolic profile during obesity. Thirty‐nine patients suffering from severe obesity, with (*n* = 23) or without (*n* = 16) periodontitis, were enrolled. We examined the subgingival microbiota composition, periodontal status and salivary inflammatory response. The fecal microbiota composition was assessed by metagenomic analysis. Inflammatory and metabolic markers were measured in the plasma and epiploon visceral adipose tissue collected during bariatric surgery. Results show that patients with periodontitis exhibited an oral microbiota dysbiosis characterized by an increased abundance of bacteria from the red and orange complexes, worsened periodontal parameters (plaque index, bleeding index, gingival recession, probing depth and clinical attachment level), and higher IL‐6 salivary levels. In fecal samples of patients with periodontitis, a higher proportion of the Proteobacteria phylum and changes in functional profile of bacteria were detected. Periodontitis was also linked to higher circulating concentrations of anti‐ *P. gingivalis* IgG, total cholesterol and lipoprotein (a). Moreover, periodontitis was associated with an enhanced production of TLR2, MyD88 and TGFβ, as well as higher activities of SOD and catalase antioxidant enzymes in the adipose tissue. Overall, these findings demonstrate that during obesity, the periodontal infection correlates with deregulated oral and gut microbiota composition, higher levels of pro‐inflammatory mediators, and altered markers of oxidative stress and lipid metabolism.

## Graphical abstract

In patients with severe obesity, periodontitis is associated with oral microbiota dysbiosis which influences gut microbiota composition by increasing Proteobacteria proportion. These alterations are linked to systemic immune and metabolic changes, including higher circulating levels of anti‐*Porphyromonas gingivalis* lgG and deregulated lipid profile. Concomitantly, periodontitis is associated with pro‐inflammatory markers and oxidative stress in the visceral adipose tissue. Overall, these findings suggest interactions between periodontal status, gut microbiota and adipose tissue, contributing to metabolic and inflammatory disorders in obesity.

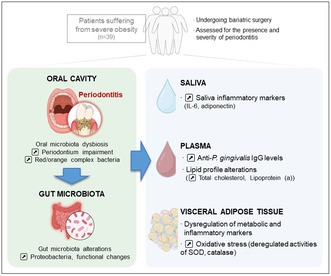

## 1 Introduction

Obesity, defined as an abnormal excessive accumulation of body fat mass, constitutes a risk factor for a wide range of complications, including insulin resistance, type 2 diabetes, and vascular disorders [1]. Moreover, obesity is associated with a change in the gut microbiota signature characterized by an altered Firmicutes‐to‐Bacteroidetes phyla ratio, a reduced taxonomic diversity, and an increased level of gram‐negative bacteria [2]. Notably, an enrichment in the Proteobacteria phylum has been linked to an elevated intestinal permeability [3] and higher plasma levels of bacterial lipopolysaccharides (LPS) reflecting metabolic endotoxemia [4]. This endotoxemia contributes to systemic low‐grade inflammation, known to promote insulin resistance [5]. Several epidemiological and clinical studies have also established a positive association between obesity, oral microbiota dysbiosis, and bacterial infections such as periodontitis [6, 7].

Periodontitis is a highly prevalent oral infectious disease characterized by a chronic inflammation of the tooth‐supporting tissue, leading to its destruction. The progression of periodontitis is marked by the growth of gram‐negative bacteria, including specific bacterial complexes identified by Socransky and co‐workers [8]. The “red complex”, consisting of *Porphyromonas gingivalis* , *Tannerella forsythia,* and *Treponema denticola* , is strongly linked to advanced periodontitis and severe tissue destruction [8, 9]. The “orange complex”, which includes species such as *Prevotella intermedia* , *Fusobacterium nucleatum,* and *Peptostreptococcus micros* , is considered a precursor of the red complex and contributes to periodontal disease progression [8, 9]. These pathogens elicit a host immune response that inadvertently results in the destruction of periodontal tissues [10]. The release of pro‐inflammatory cytokines, matrix metalloproteinases, and reactive oxygen species (ROS) leads to the degradation of periodontal ligament and alveolar bone. Growing literature data report a causal link between periodontitis and a range of systemic metabolic diseases, including metabolic syndrome, diabetes, non‐alcoholic fatty liver disease, and cardiovascular or neurodegenerative disorders [11]. Periodontitis is associated with elevated circulating levels of pro‐inflammatory markers such as C‐reactive protein (CRP) and interleukin‐6 (IL‐6) [12], and disruption in lipid metabolism [13, 14]. Importantly, periodontal treatment helps to limit these alterations [15].

Several biologically plausible mechanisms have been proposed to explain the systemic impact of periodontitis. One primary pathway involves the dissemination of oral bacteria and their products, such as LPS, into the bloodstream, contributing to systemic inflammation. In patients with periodontitis, bacteremia and endotoxemia have been shown to occur not only after professional procedures [16, 17] but also during daily oral activities such as tooth brushing and chewing [18, 19]. DNA from periodontal pathogens such as *Aggregatibacter actinomycetemcomitans* , *P. gingivalis,* and *T. denticola* has been detected in cardiovascular tissues [20, 21]. Additionally, these bacteria and their components have been identified in distant sites, such as the brain and β pancreatic islets [22, 23]. In blood vessels, *P. gingivalis* DNA and proteases detection are associated with neutrophil infiltration [24, 25]. Pro‐inflammatory mediators and immune complexes originating from the oral cavity may further contribute to systemic inflammation [26]. Emerging evidence also suggests that periodontitis may indirectly influence systemic health through disruption of the gut microbiota ecology. Given that individuals swallow 1 to 1.5 L of saliva per day [27], chronic swallowing of periodontal bacteria, cytokines, and immune cells involved in the host response during periodontitis may contribute to gut microbiota dysbiosis. Bao et al. [28] demonstrated that administrating saliva samples from periodontitis patients to healthy mice resulted in gut microbiota alterations and increased intestinal inflammation. While studies using animal models investigated the impact of periodontal infection on gut microbiota [29, 30] data obtained from clinical studies remain limited.

During obesity, the adipose tissue becomes a dysfunctional metabolic organ marked by changes in adipocyte hypertrophy, infiltration of immune cells, and increased secretion of pro‐inflammatory adipokines [31]. These changes drive chronic low‐grade inflammation and oxidative stress, which contribute to obesity‐related insulin resistance [32]. The visceral adipose tissue has received particular attention due to its strong association with metabolic complications. Arimatsu et al. [29] demonstrated that oral administration of *P. gingivalis* in mice led to alterations in gut microbiota composition, an increased abundance of Bacteroidetes, and exacerbated adipose tissue inflammation, marked by macrophage infiltration, ultimately contributing to insulin resistance. Similarly, experimental periodontitis in rodents aggravates adipose tissue inflammation via nuclear factor‐kappa B (NF‐κB) pathway [33]. Furthermore, we previously demonstrated that exposure to *P. gingivalis* LPS promotes inflammation and oxidative stress in adipocytes [34]. Although these studies provide evidence linking periodontitis to adipose tissue metabolic dysfunction, there is still a lack of data from human studies. In the context of obesity, periodontitis may exacerbate adipose tissue deregulation, thereby aggravating its metabolic dysfunction.

This study aimed to evaluate the relationships between the presence of periodontitis, oral and gut microbiota composition, and inflammatory and metabolic markers in patients with severe obesity. First, we characterized the periodontal status of patients with grade II or III obesity and assessed the extent of oral microbiota dysbiosis by analyzing the subgingival bacteria composition. Saliva samples were used to determine the local inflammatory response associated with periodontitis. Next, we explored the interplay between periodontitis and the gut microbiota composition by performing bacterial DNA metagenomic analysis of fecal samples. In parallel, blood and epiploon visceral adipose tissue collected during bariatric surgery were analyzed in order to evaluate major inflammatory and metabolic markers related to obesity.

## 2 Materials and Methods

### 2.1 Clinical Study Design

Recruitment of patients was achieved in the department of digestive surgery from La Réunion Hospital (CHU, La Réunion), after the study design was approved by the National Medical Ethics Committee (Clinical Trial N° 2016A082249) and the written informed consent was received from participants. Thirty‐nine patients suffering from grade III obesity with a body mass index (BMI) ≥ 40 kg/m^2^ or grade II obesity (BMI ≥ 35 kg/m^2^ with co‐morbidities including diabetes, hypertension, and sleep apnea, leading to eligibility for bariatric surgery) and undergoing bariatric surgery scheduled within the digestive surgery department were included. Inclusion criteria were: male or female (at least 18 years old), eligible for bariatric surgery scheduled within the digestive surgery department of CHU de La Réunion, affiliated to Social Security system or equivalent. Exclusion criteria were: treatment with steroids or anti‐inflammatory drugs in progress (or stopped for less than 1 month), patient who received antibiotics within 3 months prior to the bariatric surgery for whatever duration, patient with current dental care (or less than 3 months), person under guardianship or deprived of liberty, patient participating in another research protocol. The day before the bariatric surgery, the patients recruited were examined to evaluate the presence and grade of periodontitis (mild, moderate, and severe). Then, patients with mild, moderate, or severe periodontitis were classified in a first group called “with periodontitis” (Presence), and periodontally healthy patients placed in a second group called “without periodontitis” (Absence). Of note, the sample size calculation was based on the primary outcome of the study aiming to evidence a 20% difference in IL‐6 levels released from the adipose tissue between both groups, with a statistical power of at least 80% and a two‐sided alpha risk of 5%. The estimation also accounted for an expected patient consent rate of 80%, as well as a prevalence of moderate and severe periodontitis estimated at 33%, based on literature data [6, 35, 36]. Table 1 summarizes the description of patient population according to the presence or absence of periodontitis, with anthropometric parameters, daily dietary consumption, comorbidities, and ongoing medical treatments.

**TABLE 1:** Anthropometric, nutritional, and clinical parameters of the patients according to the periodontal status.

	Absence of periodontitis	Presence of periodontitis	*p*
Gender (female/male)	13/3	21/2	0.631
Age (years)	34.20 ± 9.47	39.35 ± 9.41	0.204
BMI (kg/m^2^)^a^	45.81 ± 8.74	45.73 ± 6.91	0.621
Fat mass (%)	48.51 ± 5.41	49.49 ± 3.84	0.909
CRP (mg/L)	11.15 ± 9.50	9.88 ± 6.30	0.956
Ferritin (μg/L)	64.36 ± 58.73	120.87 ± 114.77	0.063
*Dietary consumption*			
Calories (kcal/day)	1658.17 ± 316.31	1592.18 ± 173.15	0.671
Proteins (g/day)	90.67 ± 19.84	85.29 ± 12.14	0.922
Fibers (g/day)	18.08 ± 9.61	17.00 ± 4.56	0.918
Carbohydrates (g/day)	193.00 ± 39.35	151.65 ± 86.32	0.376
Iron (mg/day)	8.74 ± 2.08	7.72 ± 1.30	0.377
Calcium (ng/day)	737.75 ± 199.01	698.00 ± 228.41	0.631
*Comorbidities (n)*			
Diabetes (T2D/T1D)^b^	2/1 (20.0%)	3/0 (13.0%)	1
Dyslipidemia	0 (0.0%)	3 (13.0%)	0.264
Hypertension	3 (20.0%)	11 (47.8%)	0.101
Sleep apnea	8 (53.3%)	15 (65.2%)	0.514
Hepatic steatosis	12 (80.0%)	17 (73.9%)	1
*Treatments (n)*			
Metformin	2 (13.3%)	4 (17.4%)	1
Other OAD^c^	2 (13.3%)	2 (8.7%)	1
Insulin	1 (6.7%)	2 (8.7%)	1
GPL‐1 analog	1 (6.7%)	1 (4.4%)	1
Antihypertensive	2 (13.3%)	8 (34.8%)	0.259
Hypolipidemic	0 (0.0%)	4 (17.4%)	0.139

*Note:* Patients with obesity were enrolled in the study and classified as with (Presence, *n* = 23) or without (Absence, *n* = 16) periodontitis. Data are expressed as mean ± standard deviation for quantitative data and *n* (%) for categorical data. *p* Values refer to comparisons between the two groups using Mann–Whitney test or Fisher's exact test when appropriate.

^a^ BMI measured on the day of bariatric surgery.

^b^ T2D for type 2 diabetes, T1D for type 1 diabetes.

^c^ OAD for oral antidiabetics.

### 2.2 Characterization of Periodontal Status

During the periodontal examination performed by the dental surgeon at the hospital (CHU, La Réunion), all teeth except the 3rd molars were examined at 6 sites, including the mesio‐buccal, mid‐buccal, disto‐buccal, mesio‐lingual, mid‐lingual, and disto‐lingual sites, using a millimeter periodontal probe PCB‐UNC 15 (Hu Friedy, Hu Friedy Group, Markham, ON, Canada). For each site, 5 clinical indicators, namely plaque index, bleeding index, gingival recession, probing depth (PD), and clinical attachment level (CAL), were recorded. For each tooth, scores of all examined surfaces were summed and divided by the number of examined surfaces. Results were expressed as average per tooth.

For the evaluation of the plaque index, the gradation from Silness & Löe [37] was used, as follows: 0 = no microbial plaque, 1 = thin film of microbial plaque adhering to the free gingival margin which is only detectable using the probe, 2 = moderate accumulation of microbial plaque in the sulcus (on the tooth or gingival margin) visible to the naked eye, 3 = abundance of plaque along the tooth and gingival margin.

For the evaluation of the bleeding index, the gradation from Silness and Löe [38] was used, as follows: 0 = normal gingival, no bleeding on probing, 1 = no bleeding on probing, slight changes in color and texture, mild inflammation, 2 = bleeding on probing, moderate inflammation, redness, edema. 3 = tendency to spontaneous bleeding, severe inflammation, marked redness, edema, and possible ulceration.

The gingival recession was measured in mm from the cemento‐enamel junction to the depth of the free gingival margin using a millimeter periodontal probe.

The PD was measured in mm from the gingival margin to the base of the periodontal pocket using a millimeter periodontal probe.

The CAL, defined as the distance from the base of the pocket to the cementoenamel junction of the tooth, was calculated by adding PD and recession values.

Periodontal status of patients was defined in accordance with the American Academy of Periodontology/Centers for Disease Control and Prevention case definition [35]. This led to identify (i) patients without periodontitis, (ii) patients with mild periodontitis characterized by at least 2 proximal sites with attachment loss of ≥ 3 mm, and 2 different teeth with periodontal pocket depths ≥ 4 mm or 1 site with pocket depths ≥ 5 mm, (iii) patients with moderate periodontitis characterized by at least 2 proximal sites with attachment loss of ≥ 4 mm or 2 different teeth with periodontal pocket depths ≥ 5 mm, and (iv) patients with severe periodontitis characterized by at least 2 proximal sites with attachment loss of ≥ 6 mm on 2 different teeth and ≥ 1 site with pocket depths ≥ 5 mm.

### 2.3 Sample Collection

Samples of periodontal pocket fluid were collected in patients during the periodontal examination performed by the dental surgeon at the hospital (CHU, La Réunion) on the day before the bariatric surgery using a commercial Perio‐Analyze kit (Clinident Institute, Aix‐en‐Provence, France). Sterile paper points were inserted into the deepest periodontal pocket of 4 different teeth for 15 s to collect the pocket fluid and were pooled in a sterile tube. All samples were then sent to the Clinident Institute for quantification of total flora and 9 periodontal bacteria species, including *A. actinomycetemcomitans* , *P. gingivalis* , *T. forsythia* , *T. denticola* , *P. intermedia* , *F. nucleatum* , *P. micros* , *Campylobacter rectus,* and *Eikenella corrodens* by PCR test. This methodological approach routinely employed in dental practice for detection of key pathogens allowed evaluating periodontal bacterial dysbiosis using markers that are directly relevant for clinical periodontal assessment. In parallel, a volume of 10 mL of stimulated saliva was collected in a sterile polypropylene tube and stored at −80°C until analysis.

Stool samples (50 g) were collected according to the International Human Microbiome standard. After homogenization, samples were frozen using liquid nitrogen and stored at −80°C until analysis.

Blood sampling was performed just before the bariatric surgery. Briefly, blood samples (10 mL) were collected from the antecubital vein of fasting patients using EDTA vacutainer tubes. After centrifugation at 2000 *g* for 15 min at room temperature, plasma was recovered and stored at −80°C until analysis.

Epiploon visceral adipose tissue samples (50 g) were collected during the bariatric surgery and placed in a sterile polypropylene tube. Samples were weighed, snap‐frozen in liquid nitrogen, and stored at −80°C until analysis.

### 2.4 Adipose Tissue Protein Extraction and Quantification

Epiploon visceral adipose tissue samples (100 mg) were homogenized in 600 μL of lysis buffer (Tris–HCl (20 mm) pH 8.5, EDTA (1 mm), Triton X‐100 (0.05%), pH 7.4). Then, tissues were lysed by TissueLyser II (Qiagen, Courtaboeuf, France) with 2 tungsten carbide balls per tube at 30 Hz for 1 min, and centrifuged at 14 000 *g* for 10 min at 4°C. Supernatants containing proteins were collected for protein quantification by the bicinchoninic acid assay [39] and used for ELISA as well as enzymatic activity assays.

### 2.5 Quantification of Inflammatory and Metabolic Markers in Saliva, Plasma, and Adipose Tissue

Saliva, plasma and adipose tissue protein lysates were analyzed by ELISA kits targeting human interleukins (IL‐6, IL‐8, IL‐10), tumor necrosis factor‐alpha (TNF‐α), monocyte chemoattractant protein‐1 (MCP‐1, eBioscience, ThermoFisher Scientific, Dardilly, France), adiponectin, resistin (Ray Biotech, USA), leptin, myeloperoxidase (MPO), LPS‐binding protein (LBP), lipoprotein(a) (Lp(a), Abcam, France) and proprotein convertase subtilisin/kexin type 9 (PCSK9, R&D Sytems, Abingdon, UK). Levels of serum triglycerides, total cholesterol, high‐density lipoprotein (HDL)‐cholesterol, and glycemia were measured by Hospital Autoanalyzer (Dade Behring Inc., Deerfield, IL, USA) at CHU de La Réunion through enzymatic methods. Low‐density lipoprotein (LDL)‐cholesterol was calculated according to Friedewald equation. Plasma levels of insulin and CRP were quantified by Hospital Autoanalyzer at CHU de La Réunion by immunoassays. Concerning adipose tissue samples, concentrations of targeted proteins were normalized to total tissue protein contents.

### 2.6 Quantification of Immunoglobulin G (IgG) Antibody Against *P. gingivalis* in Saliva and Plasma

*P. gingivalis* (American Type Culture Collection ATCC‐33277, Pasteur Institute Collection, Paris, France) was cultured into 2.1% mycoplasma broth base supplemented with 5 μg/mL of hemin and 1 μg/mL of menadione (Sigma‐Aldrich, Saint‐Louis, MO, USA) at 37°C in an anaerobic environment (GENbag anaer, bioMérieux, Craponne, France). Then, bacteria were formalin‐fixed and used as antigens to detect saliva and plasma IgG antibody by ELISA, according to the method previously published [40]. Briefly, *P. gingivalis* bacterial suspension was prepared in Phosphate Buffer Saline (PBS) to obtain an optical density of 0.15 arbitrary unit (AU) at 580 nm (CLARIOstar Plus, BMG Labtech, Aylesbury, UK). A volume of 100 μL/well of bacterial suspension was incubated overnight at room temperature in a Nunc Maxisorp 96‐well plate to allow bacteria attachment to the solid phase. The ELISA assay was performed with 100 μL of patient saliva or plasma sample per well, at an appropriate dilution in PBS supplemented with fetal bovine serum, and developed with polyclonal goat anti‐human IgG conjugated with horseradish peroxidase (1:20 000, ZyMax, VWR, Rosny‐sous‐Bois, France). Absorbance was measured at 450 nm (CLARIOstar Plus), and IgG levels were expressed as AU.

### 2.7 Measurement of Antioxidant Enzyme Activities

Adipose tissue protein lysates were used for the measurement of superoxide dismutase (SOD), catalase, and glutathione peroxidase (GPx) enzymatic activities, according to the method previously published [41]. Total SOD activity was determined by monitoring the rate of acetylated cytochrome c reduction by superoxide radicals generated by the xanthine/xanthine oxidase system. Measurements were performed in a reagent buffer (xanthine oxidase, xanthine (0.5 mM), cytochrome c (0.2 mM), KH_2_PO_4_ (50 mM), EDTA (2 mM), pH 7.8) at 25°C. Assays were monitored using spectrophotometry at 560 nm (CLARIOstar Plus). For specific manganese‐SOD (MnSOD) activity measurement, samples were incubated with NaCN (1 mM) to inhibit the copper/zinc‐SOD (Cu/ZnSOD) activity. Total SOD, MnSOD, and resulting Cu/ZnSOD activities were expressed as international units per μg of protein.

Catalase activity was assessed based on the ability of the catalase enzyme to convert hydrogen peroxide into dioxygen and water. The blank was measured at 240 nm before adding hydrogen peroxide (10 mM final concentration) to start the reaction. Catalase activity was determined by measuring the absorbance at 240 nm and expressed as international units per μg of protein.

Total GPx activity was evaluated using cumene hydroperoxide as substrate. The rate of glutathione oxidized by cumene hydroperoxide (6.5 mM) was evaluated by the reduction of NADPH (0.12 mM), in the presence of a tris buffer (50 nM, pH 8) containing NaCN (10 mM), reduced glutathione (0.25 mM), and glutathione reductase (1 U/mL). The GPx activity was measured by monitoring the absorbance at 340 nm. The rate of enzymatic activity was expressed as international units per μg of protein.

### 2.8 Adipose Tissue Histomorphometry

Frozen adipose tissue samples were fixed in formaldehyde and embedded in paraffin. Sections (4 μm/section) were performed and stained with Picrosirius Red as described previously [42]. Adipocyte size was determined as the mean cell area of 100 intact adipocytes/subject on digital images acquired at 40× magnification with an ECHO microscope revolve R3/R4 (Discover Echo, San Diego, CA, USA) under white light using ImageJ software. Total fibrosis was quantified on digital images acquired at 100× magnification under polarized light as Picrosirius Red stained tissue area for 100 adipocytes using ImageJ software.

### 2.9 Adipose Tissue RNA Extraction

Frozen adipose tissue (100 mg) was disrupted and homogenized in 1 mL of QIAzol Lysis Reagent, and extraction of total RNA was performed according to the manufacturer's instructions (RNeasy Lipid Tissue Mini Kit, Qiagen). Briefly, after 5 min incubation, the homogenate was mixed with chloroform and centrifuged at 12 000× *g* for 15 min at 4°C. The upper aqueous phase was collected in a new tube and mixed with an equal volume of 70% ethanol. Next, the sample was transferred to RNeasy Mini spin column, and the RNA fraction was eluted in 40 μL of RNAse‐free water. Concentration and purity of RNA were determined using SPECTROstar Nano (BMG Labtech).

### 2.10 Quantitative Polymerase Chain Reaction (qPCR) Analysis

Total RNA (1 μg) isolated from frozen adipose tissue was reverse transcribed using Random hexamer primers (Invitrogen, ThermoFisher Scientific). The qPCR was performed using SYBRGreen master mix (Sigma‐Aldrich) and 10 μM primers (Eurogentec, Liège, Belgium). Analysis of genes encoding F4/80, cluster of differentiation (CD)36, CD14, Toll‐like receptor (TLR)2, TLR4, TLR9, myeloid differentiation primary response 88 (MyD88), NF‐κB, cyclooxygenase‐2 (COX‐2), inducible nitric oxide synthase (iNOS), NADPH oxidase (NOX)4, NOX2, GPx, Cu/ZnSOD, MnSOD, catalase, nuclear factor erythroid 2‐related factor 2 (Nrf2), fatty acid synthase (FAS), adipose triglyceride lipase (ATGL), hormone‐sensitive lipase (HSL), lipoprotein lipase (LPL), transforming growth factor β (TGFβ) and fibronectin (FN1) was performed. Relative expression of targeted genes was normalized to the β‐actin gene expression, according to the 2^−ΔΔCT^ method [43]. Primer sequences are listed in Table 2.

**TABLE 2:** Primers used for RT‐qPCR analysis.

Gene	Forward	Reverse
*β‐actin*	ACC‐TTC‐TAC‐AAT‐GAG‐CTG‐CG	CCT‐GGA‐TAG‐CAA‐CGT‐ACA‐TGG
*F4/80*	CCC‐TCA‐GCA‐AAT‐ATC‐ACT‐CCG	CAC‐CCG‐ATC‐TTC‐ATC‐TTA‐TCC‐C
*CD14*	AAG‐ACT‐TAT‐CGA‐CCA‐TGG‐AGC	CGG‐CAT‐GGA‐TCT‐CCA‐CCT‐C
*CD36*	AGA‐TGC‐AGC‐CTC‐ATT‐TCC‐AC	CGT‐CGG‐ATT‐CAA‐ATA‐CAG‐CA
*TLR4*	TCT‐ACA‐AAA‐TCC‐CCG‐ACA‐AC	TGG‐ATT‐TCA‐CAC‐CTG‐GAT‐A
*TLR2*	AGC‐ACT‐GGA‐CAA‐TGC‐CAC‐ATA‐C	CAT‐TGC‐GGT‐CAC‐AAG‐ACA‐GAG‐A
*TLR9*	GCA‐GAG‐CGC‐AGT‐GGC‐AGA‐CT	TGG‐GCC‐AGC‐ACA‐AAC‐AGC‐GT
*MyD88*	AAC‐TGC‐AGA‐CAC‐AAG‐CGG‐AC	CTG‐CAC‐AAA‐CTG‐GAT‐GTC‐GC
*NF‐κB*	GAA‐CCA‐CAC‐CCC‐TGC‐ATA‐TAG	GCA‐TTT‐TCC‐CAA‐GAG‐TCA‐TCC
*COX‐2*	ATC‐ACA‐GGC‐TTC‐CAT‐TGA‐CC	CAG‐GAT‐ACA‐GCT‐CCA‐CAG‐CA
*iNOS*	CCC‐ACA‐GAG‐TCG‐GCA‐CTC	TGG‐ATG‐CAA‐CCC‐CAT‐TGT‐C
*NOX4*	AGT‐CAA‐ACA‐GAT‐GGG‐ATA	TGT‐CCC‐ATA‐TGA‐GTT‐GTT
*NOX2*	CCA‐GTG‐AAG‐ATG‐TGT‐TCA‐GCT	GCA‐CAG‐CCA‐GTA‐GAA‐GTA‐GAT
*GPx*	CCA‐AGC‐TCA‐TCA‐CCT‐GGT‐CT	TCG‐ATG‐TCA‐ATG‐GTC‐TGG‐AA
*Cu/ZnSOD*	GGA‐GGT‐GTG‐GGG‐AAG‐CAT‐TA	ACA‐TTG‐CCC‐AAG‐TCT‐CCA‐AC
*MnSOD*	CGT‐CAC‐CGA‐GGA‐GAA‐GTA‐CC	CTG‐ATT‐TGG‐ACA‐AGC‐AGC‐AA
*Catalase*	CGT‐GCT‐GAA‐TGA‐GGA‐ACA‐GA	AGT‐CAG‐GGT‐GGT‐GGA‐CCT‐CAG‐TG
*Nrf2*	GAG‐AGC‐CCA‐GTC‐TTC‐ATT‐GC	TGC‐TCA‐ATG‐TCC‐TGT‐TGC‐AT
*ATGL*	TGG‐ATG‐TTG‐GTG‐GAG‐CTG‐TC	TGG‐ATG‐TTG‐GTG‐GAG‐CTG‐TC
*FAS*	AGA‐CAC‐TCG‐TGG‐GCT‐ACA‐GCA‐T	ATG‐GCC‐TGG‐TAG‐GCG‐TTC‐T
*HSL*	CCC‐TCA‐TGG‐CTC‐AAC‐TCC‐TTC‐C	TTG‐ACA‐TCG‐GAG‐GGT‐GTG‐GAG‐G
*LPL*	GGT‐CGA‐AGC‐ATT‐GGA‐ATC‐CAG	TAG‐GGC‐ATC‐TGA‐GAA‐CGA‐GTC
*TGFβ*	GCC‐TTT‐CCT‐GCT‐TCT‐CAT‐GG	TCC‐TTG‐CGG‐AAG‐TCA‐ATG‐TAC
*FN1*	ACT‐GTA‐CAT‐GCT‐TCG‐GTC‐AG	AGT‐CTC‐TGA‐ATC‐CTG‐GCA‐TTG

### 2.11 Fecal Bacterial DNA Extraction, Library Preparation, and Microbiota Sequencing

Fecal bacterial DNA was extracted from 200 mg of stool sample, using “Pure Link Microbiome DNA Purification Kit” (Invitrogen), according to an optimized protocol previously developed [44]. DNA quality was assessed using NanoDrop (ThermoFisher Scientific), and DNA concentration determined by a Qubit fluorometer (Invitrogen). To build the library, 1.5 μg of DNA was used. NEBNext Ultra II End Repair/dA‐Tailing Module from New England Biolabs (NEB, Évry, France) was used for performing “end‐prep” step. Then, 1D Native Barcoding Genomic DNA Kit (Oxford Nanopore Technologies (ONT), Oxford, UK) and “NEB Blunt/TA Ligase Master Mix kit” (NEB) were used for DNA multiplexing and adapter ligation, respectively. DNA purification was achieved using Agentcourt AMPure XP beads (Beckman Coulter, Brea, CA, USA). Whole‐genome metagenomic sequencing was performed with ONT's MinION sequencer using 48 h runs. For each run, 12 samples were simultaneously loaded on flow cells.

### 2.12 Bioinformatic Analysis of Fecal Microbiota

Analysis was performed as described previously [44]. Briefly, after base‐calling, demultiplexing, and quality filtration steps, Nanopore reads were first taxonomically binned using Centrifuge. Subsequently, read bins were aligned against the corresponding reference from the Unified Human Gastrointestinal Collection (UHGC)1.0 genome using Minimap2, keeping read‐genome pairs supported by a mapping quality ≥ 5. From these results, species‐level abundance tables were computed.

Alpha diversities (species richness and Shannon index) were determined from rarefied species‐level abundance table with the phyloseq R package [45]. For samples not reaching the fixed threshold, an upsizing procedure was applied to estimate diversity based on regression models of diversity at consecutive downsizing levels.

For the functional profiling, an abundance table of KEGG orthology groups (KO groups) was built from the species‐level abundance table by computing the sum of the species harboring these KO groups. The KO abundance was next summarized in the form of the abundance of Gut Microbiome Modules (GMM) with GOmixer R package [46].

### 2.13 Analysis of Bacterial DNA From the Adipose Tissue

For bacterial DNA extraction from the adipose tissue, the same method as described above for fecal samples was carried out, with the addition of a delipidation step for the tissue lysate before the addition of ammonium acetate. All experiments were performed in a sterile environment. A negative control sample of DNA‐free water was run in each set of experiments. Whole‐genome metagenomic sequencing was performed with ONT's MinION sequencer using 48 h runs. For each run, 11 samples of DNA extracted from the adipose tissue and 1 negative control sample were simultaneously loaded on flow cells. A total of 37 adipose tissue samples and 4 negative control samples were analyzed. In brief, 14.04 million reads were generated, with an average of 37 705 reads per run per adipose tissue sample, and 16 866 reads per blank sample. A large fraction of the reads was from human origin (98.36%). Once excluded the human reads, on average 6041 non‐human reads for adipose tissue samples and 364 non‐human reads for blank samples were retained. Analysis was then performed as described above. Comparing the species detected in the abundance table computed, 39 species were detected in the adipose tissue samples and 16 in blank samples, of which 12 species were shared between both types of samples. These 12 species were considered contaminants and excluded from following analysis of adipose tissue samples. Contaminant detection was carried out in the remaining 27 species with the DECONTAM R package, which computed two different methods based on frequency and prevalence to identify potential contaminants in metagenomic analysis. We used the function *isNotContaminant* to identify non‐contaminant sequence features in very low biomass samples. *isNotContaminant* implements the prevalence method, with the standard prevalence score P replaced with 1‐P, given that low scores are those associated with non‐contaminants. Results were limited to the 9 species only detected in adipose tissue samples and with status non‐contaminant by DECONTAM.

### 2.14 Statistical Analysis

For most of the following results, patients were divided into 2 groups: Absence (patients without periodontitis) and Presence (gathering patients with mild, moderate, and severe periodontitis). For the analysis of selected markers from the visceral adipose tissue, including LBP levels, adipocyte and fibrosis area, and mRNA levels of inflammatory and metabolic markers, comparisons were performed between patients without periodontitis and patients with moderate periodontitis. Balanced sample sizes (*n* = 12) and comparable age and sex distributions were achieved for these groups. Data were expressed as the mean ± standard deviation (SD) for quantitative data or as *n* (%) for categorical data. Variables were tested for normality, then comparison between groups was assessed using the Mann–Whitney test for continuous variables or Fisher's Exact test for discrete and categorical data, as appropriate. Spearman test was used for non‐parametric assessment of correlations. Two‐sided tests were used and significant differences were considered for a *p*‐value < 0.05. Statistical analysis was carried out with SAS version 9.4 (Institute Inc., Cary, NC, USA), STATA version 16.1 (StataCorp LLC, College Station, TX, USA) or Prism version 9 (GraphPad Software Inc., San Diego, CA, USA), as appropriate.

## 3 Results

### 3.1 Anthropometric, Nutritional, and Clinical Parameters of the Patients

A total of 39 participants with obesity were enrolled in the study (Table 1), with 41% classified as without periodontal infection (*n* = 16) and 59% diagnosed with periodontitis (*n* = 23). Both groups presented grade II or III obesity, as indicated by BMI values of 45.81 ± 8.74 kg/m^2^ in the non‐periodontitis group and 45.73 ± 6.91 kg/m^2^ in the periodontitis group. No significant difference was observed between the two groups in terms of gender distribution, age, fat mass, and CRP circulating level. In addition to anthropometric assessments, daily intake of calories, proteins, fibers, carbohydrates, and minerals showed no significant variation between the two groups. Furthermore, no difference was detected in the prevalence of comorbidities or ongoing medical treatments between groups. The prevalence of metabolic and cardiovascular comorbidities, including diabetes, dyslipidemia, hypertension, sleep apnea, and hepatic steatosis, was not significantly different between the two groups, despite only patients with periodontitis exhibited dyslipidemia as a comorbidity factor (13%) and received a lipid‐lowering treatment (17%).

### 3.2 Link Between Periodontitis and Periodontal Health Markers

Periodontal health was assessed by measuring several parameters, including plaque index, bleeding index, gingival recession, PD, and CAL. These parameters were significantly altered in the periodontitis group compared to the periodontally healthy group (Table 3). In particular, plaque index and bleeding index were twice as high in patients with periodontitis, while gingival recession was elevated by a 3‐fold factor in patients with periodontal infection. No significant difference was observed between the two groups regarding the number of teeth, the score of Decayed, Missing due to caries and Filled Teeth (DMFT), smoking status, and oral care habits.

**TABLE 3:** Clinical parameters related to periodontal health, smoking status, and oral care habits of the patients.

	Absence of periodontitis	Presence of periodontitis	*p*
Plaque index	0.45 ± 0.56	0.89 ± 0.64	0.030*
Bleeding index	0.24 ± 0.31	0.59 ± 0.43	0.001**
Gingival recession (mm)	0.03 ± 0.05	0.10 ± 0.12	0.022*
Probing depth [PD] (mm)	1.21 ± 0.22	1.80 ± 0.40	< 0.001***
Clinical attachment level [CAL] (mm)	1.22 ± 0.23	1.87 ± 0.38	< 0.001***
Number of teeth (/28)	25.63 ± 3.36	24.39 ± 3.59	0.245
DMFT score^a^	11.63 ± 5.86	13.91 ± 6.15	0.346
Smoking history (*n*)			1
Never	10 (62.5%)	14 (60.9%)	
Former/Actual	6 (37.5%)	9 (39.1%)	
Toothbrushing frequency (*n*)			0.726
≥ 2 times per day	12 (75.0%)	15 (65.2%)	
1 time per day	4 (25.0%)	8 (34.8%)	
Additional oral care (dental floss, mouthwash)			0.318
Yes	8 (50.0%)	7 (30.4%)	
No	8 (50.0%)	16 (69.6%)	

*Note:* Data are expressed as mean ± standard deviation for quantitative data and *n* (%) for categorical data from patients with (Presence, *n* = 23) or without (Absence, *n* = 16) periodontitis. *p* Values refer to comparisons between the two groups using Mann–Whitney test or Fisher's exact test when appropriate.

^a^ DMFT for decayed, missing due to caries and filled teeth (sum in the permanent teeth).

* *p* < 0.05.

** *p* < 0.01.

*** *p* < 0.001 as compared to Absence of periodontitis.

### 3.3 Link Between Periodontitis and Oral Microbiota Composition

Oral microbiota composition was evaluated by quantifying 9 periodontal pathogens usually assessed in periodontal pocket fluid, using a qPCR test. The detection rates and copy numbers of these periodontal pathogens are reported on Table 4. Periodontal infection was associated with a significant increase in the total bacterial flora count. At the species level, the abundance of all evaluated bacteria was increased in patients with periodontitis, with a statistically significant difference for *P. intermedia* , *F. nucleatum,* and *C. rectus* . Interestingly, when pooled together, the count of bacteria of the red complex ( *P. gingivalis* , *T. forsythia,* and *T. denticola* ) and the number of bacteria of the orange complex ( *P. intermedia* , *P. micros,* and *F. nucleatum* ) were significantly higher in patients with periodontitis than in the periodontally healthy group. *A. actinomycetemcomitans* was detected only in patients with periodontitis (22.7%). While *P. micros* and *F. nucleatum* were detected in all subjects, the prevalence of *P. gingivalis* , *T. forsythia* , *T. denticola* , *C. rectus,* and *E. corrodens* was higher in the periodontitis group, although the differences were not statistically significant. *P. intermedia* was significantly more enriched in the periodontitis group. These results suggest that the patients suffering from periodontitis exhibited an oral microbiota dysbiosis.

**TABLE 4:** Link between the periodontal status, bacteria count, and detection rate in the periodontal pockets.

Pathogens	Bacteria count			Detection rate		
	Absence of periodontitis	Presence of periodontitis	*p*	Absence of periodontitis	Presence of periodontitis	*p*
Total bacteria count	3.77E+08 ± 7.83E+08	2.16E+09 ± 4.04E+09	0.008**			
*Porphyromonas gingivalis*	1.24E+06 ± 2.48E+06	8.52E+06 ± 1.74E+07	0.063	7 (43.7%)	15 (68.2%)	0.188
*Tannerella forsythia*	8.63E+05 ± 2.92E+06	1.63E+06 ± 2.96E+06	0.126	13 (81.3%)	20 (90.9%)	0.632
*Treponema denticola*	2.30E+06 ± 5.92E+06	1.10E+07 ± 2.41E+07	0.187	14 (87.5%)	18 (81.8%)	1
*Prevotella intermedia*	1.57E+06 ± 4.56E+06	1.33E+07 ± 4.25E+07	0.002**	12 (75.0%)	22 (100%)	0.025*
*Peptostreptococcus micros*	9.99E+05 ± 1.11E+06	3.34E+06 ± 5.87E+06	0.302	16 (100%)	22 (100%)	NA
*Fusobacterium nucleatum*	1.52E+06 ± 9.33E+05	4.17E+06 ± 4.38E+06	0.048*	16 (100%)	22 (100%)	NA
*Campylobacter rectus*	4.59E+05 ± 7.55E+05	2.59E+06 ± 5.63E+06	0.015*	15 (93.8%)	22 (100%)	0.421
*Eikenella corrodens*	3.97E+04 ± 5.74E+04	8.26E+05 ± 2.19E+06	0.145	11 (68.8%)	19 (86.4%)	0.243
*Aggregatibacter actinomicetemcomitans*	0.0 ± 0.0	6.13E+06 ± 2.80E+07	NA	0 (0.0%)	5 (22.7%)	0.061
Red complex^a^	4.40E+06 ± 9.07E+06	2.11E+07 ± 4.28E+07	0.036*			
Orange complex^b^	3.55E+06 ± 5.32+E06	2.01E+07 ± 4.65E+07	0.010*			

*Note:* Periodontal pocket fluid was obtained from patients with (Presence, *n* = 23) or without (Absence, *n* = 16) periodontitis and analyzed to detect and quantify 9 periodontal pathogens using qPCR test. Data are expressed as *n* (%) for detection rate data and as mean ± standard deviation for bacteria count. *p* Values refer to comparisons between the two groups using Fisher's exact test for detection rate and Mann–Whitney test for bacteria count.

Abbreviation: NA, not applicable.

^a^ Red complex: sum of *Porphyromonas gingivalis* , *Tannerella forsythia* and *Treponema denticola* count.

^b^ Orange complex: sum of *Prevotella intermedia* , *Peptostreptococcus micros* and *Fusobacterium nucleatum* count.

* *p* < 0.05.

** *p* < 0.01.

### 3.4 Link Between Periodontitis and Salivary Inflammatory Profile

To assess the relationship between periodontal infection and oral inflammatory response, the salivary levels of IL‐6, MCP‐1, TNFα, IL‐8, leptin, resistin, and adiponectin were measured. An Oral Inflammatory Score Composite (OISC) was calculated by summing IL‐6, MCP‐1, TNFα, and IL‐8 salivary levels. We also evaluated the immune response against the major periodontal pathogen *P. gingivalis* by measuring the salivary levels of anti‐ *P. gingivalis* IgG (Table 5). No significant difference was observed concerning the levels of MCP‐1, TNFα, IL‐8, leptin, and anti‐ *P. gingivalis* IgG and OISC between patients with or without periodontitis. Resistin levels tended to be higher in patients with periodontitis. Data show a statistically significant increase in IL‐6 and adiponectin levels in periodontitis patients, suggesting a deregulated salivary inflammatory profile during periodontitis.

**TABLE 5:** Link between the periodontal status and levels of salivary inflammatory markers.

Parameters	Absence of periodontitis	Presence of periodontitis	*p*
IL‐6 (pg/mL)	8.41 ± 9.01	19.12 ± 32.82	0.035*
MCP‐1 (pg/mL)	210.21 ± 375.46	371.04 ± 583.53	0.476
TNFα (pg/mL)	7.72 ± 6.40	8.19 ± 8.98	0.871
IL‐8 (ng/mL)	1.25 ± 1.90	0.95 ± 1.40	0.838
Leptin (ng/mL)	10.82 ± 20.84	28.79 ± 56.50	0.995
Resistin (ng/mL)	55.12 ± 88.58	119.78 ± 171.45	0.095
Adiponectin (ng/mL)	2.48 ± 2.23	10.19 ± 11.46	0.010*
OISC (pg/mL)^a^	1473.16 ± 2206.71	1344.57 ± 1721.68	0.990
Anti‐ *Porphyromonas gingivalis* IgG level (AU)	0.97 ± 0.81	0.93 ± 0.72	0.944

*Note:* Saliva samples were obtained from patients with (Presence, *n* = 23) or without (Absence, *n* = 16) periodontitis. Salivary levels of IL‐6, MCP‐1, TNFα, IL‐8, leptin, resistin, adiponectin and anti‐ *Porphyromonas gingivalis* IgG were assessed by ELISA. Data are expressed as mean ± standard deviation. *p* Values refer to comparisons between the two groups using Mann–Whitney test.

Abbreviation: AU, arbitrary unit.

^a^ OISC for oral inflammatory score composite.

* *p* < 0.05.

### 3.5 Link Between Periodontitis and Gut Microbiota Composition

**FIGURE 1:**
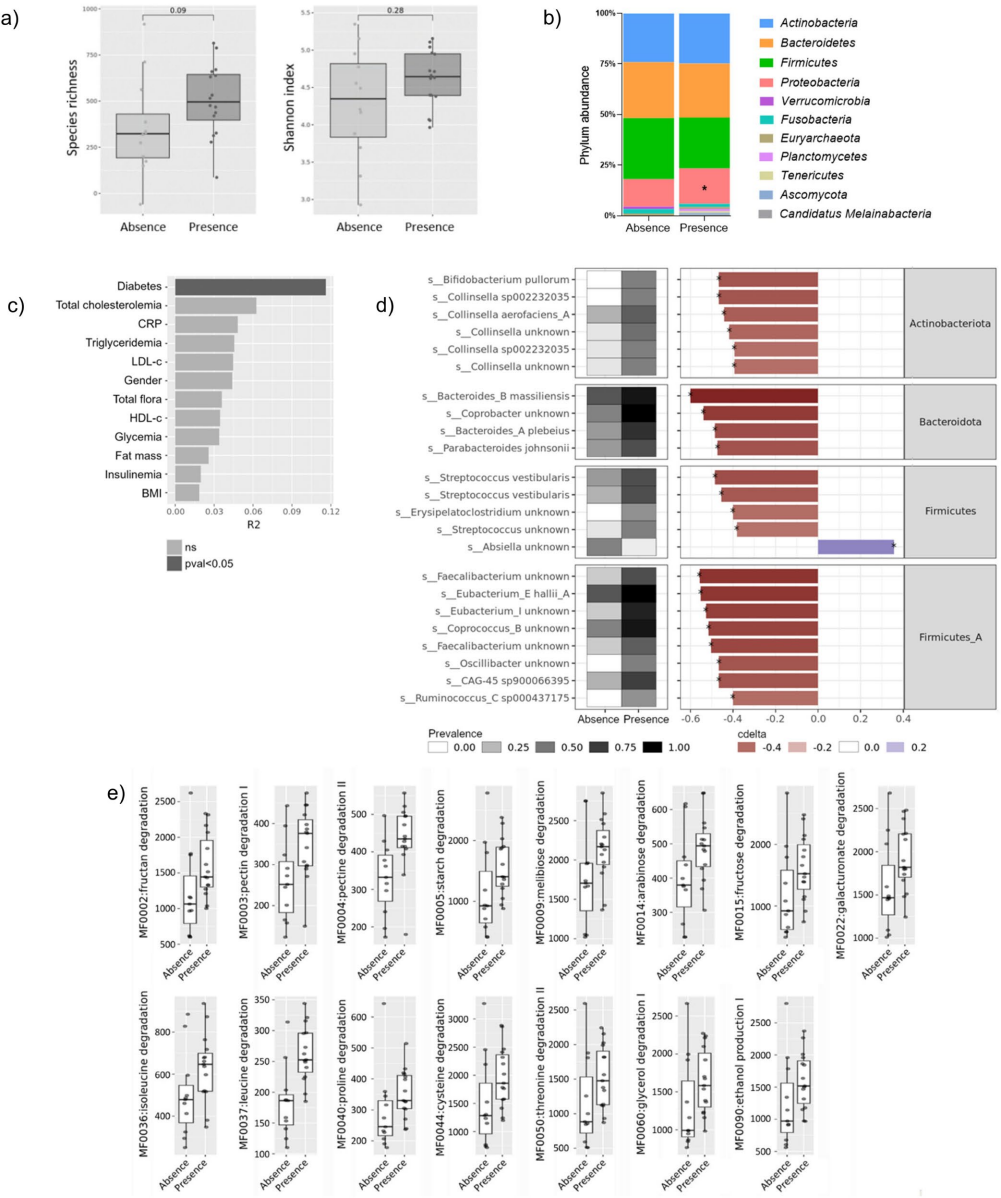
Link between the periodontal status and the taxonomic and functional profile of fecal bacterial microbiota. Metagenomic analysis was performed on DNA extracted from fecal samples obtained from patients with (Presence, *n* = 15) or without (Absence, *n* = 13) periodontitis, noting that samples were unavailable for 8 patients with periodontitis and 3 patients without periodontal infection. Comparisons for species richness and Shannon index (a) and phylum relative abundance (b) were performed by Wilcoxon rank‐sum test (*p value < 0.05). PERMANOVA test to evaluate the effect of clinical covariates on the fecal microbiome composition was assessed (c). At species level, a heatmap of 23 UHGC species‐level representatives with significant differences between individuals with and without periodontitis, assessed by Wilcoxon rank‐sum test (*p* value < 0.05), was generated (d). Variations in abundance (cliff's delta panel, right) and prevalence (left panel) were represented. For functional profiling, analysis of Gut Microbiome Module (GMM) abundance generated from the KO groups abundance table revealed 15 GMM with significant differences between the two patient groups, assessed by Wilcoxon rank‐sum test (*p* value < 0.05) (e).

To explore whether the periodontal status was associated with the gut microbiota composition, a metagenomic analysis of bacterial DNA from fecal samples was conducted. No association was found for the gut microbiota α‐diversity assessed via species richness and Shannon index (Figure 1a). The relative abundance of the Proteobacteria phylum was significantly higher (17.5%) in the periodontitis group compared to that measured in periodontally healthy individuals (13.6%) (Figure 1b). PERMANOVA analysis performed to evaluate the association between clinical covariates and fecal microbiome composition led to identifying the diabetic status as the only significant factor influencing overall fecal microbiome composition (Figure 1c). At the species level, detailed metagenomic analysis identified 23 species (UHGC quantification species‐level representatives) that were significantly different between patients with and without periodontitis (Figure 1d). Of note, 22 of these species were significantly enriched in individuals with periodontitis. This enrichment predominantly involved Firmicutes lineages, including *Streptococcus*, *Faecalibacterium*, *Eubacterium*, and *Collinsella* from the Actinobacteria phylum. Importantly, these differences were evidenced in terms of abundance but also in terms of prevalence. At the functional level, univariate analysis revealed significant differences in the abundance of 15 gut bacteria metabolic modules (GMM) between groups with and without periodontitis (Figure 1e). All of these GMMs were enhanced in patients with periodontal infection, suggesting functional shifts in the gut microbiota. Interestingly, the patients suffering from periodontitis exhibited a significant increase in the GMMs for complex carbohydrate and amino acid degradation, as well as for glycerol and ethanol metabolism.

### 3.6 Link Between Periodontitis and Plasma Inflammatory and Metabolic Profile

**FIGURE 2:**
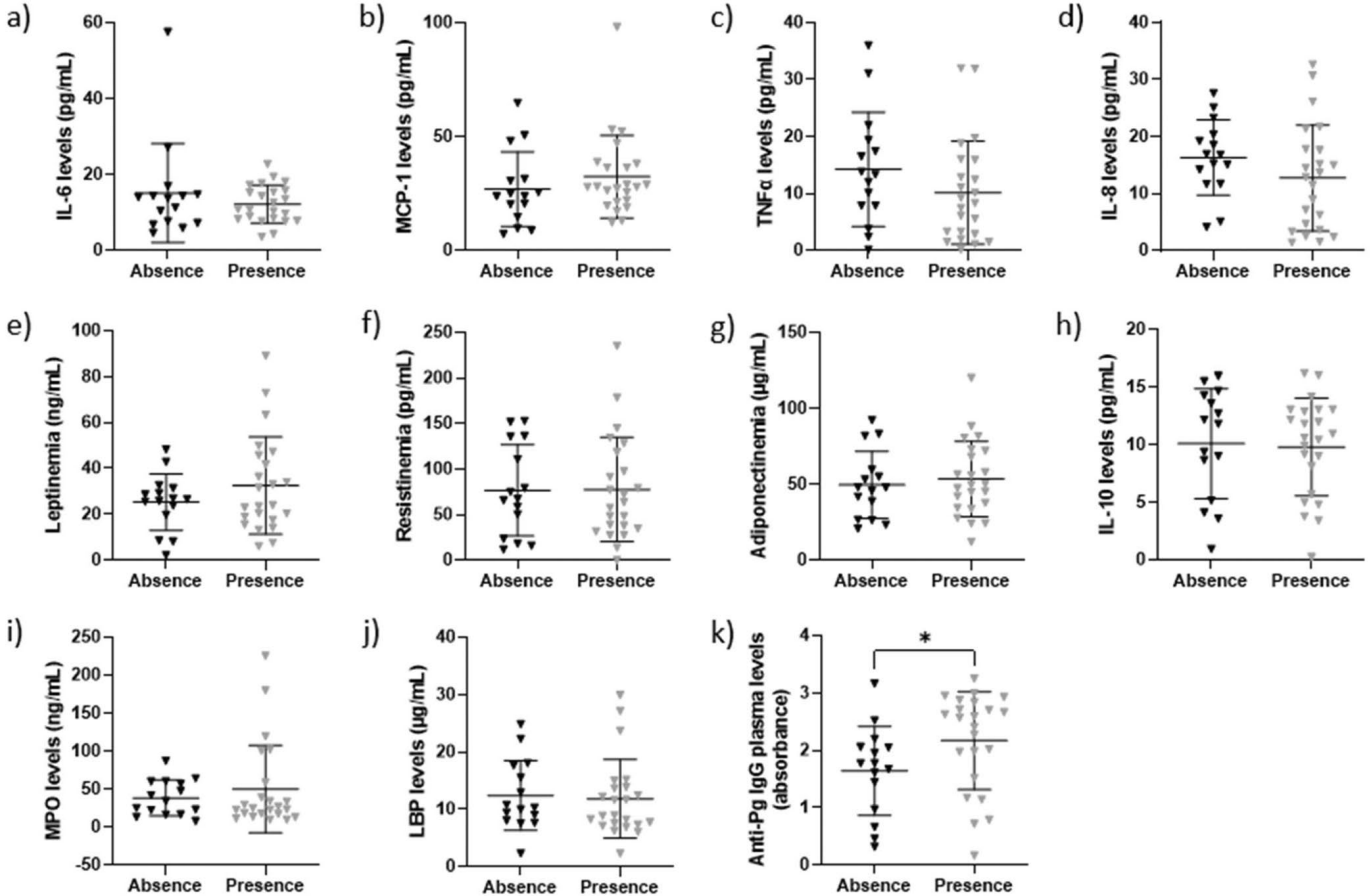
Link between the periodontal status and the levels of plasma inflammatory and metabolic markers. Blood samples were obtained from patients with (Presence, *n* = 23) or without (Absence, *n* = 13) periodontitis, noting that samples were unavailable for 3 patients without periodontal infection. Plasma levels of IL‐6 (a), MCP‐1 (b), TNFα (c), IL‐8 (d), leptin (e), resistin (f), adiponectin (g), IL‐10 (h), MPO (i), LBP (j), and anti‐ *P. gingivalis* (Pg) IgG (k) were evaluated by ELISA. Data are expressed as mean ± standard deviation. Wilcoxon rank‐sum test was performed, *p < 0.05 as compared to Absence of periodontitis.

Plasma levels of major inflammatory and metabolic markers related to obesity, and circulating concentrations of anti‐ *P. gingivalis* IgG was measured in patients with or without periodontitis. No significant difference was found for IL‐6, MCP‐1, TNFα, IL‐8, leptin, resistin, adiponectin, IL‐10, MPO and LBP (Figure 2a–j). Circulating levels of anti‐ *P. gingivalis* IgG (Figure 2k) were higher in patients with periodontitis. Data also indicate no significant difference in the circulating levels of triglycerides, HDL‐cholesterol, glucose, and insulin measured in patients with or without periodontitis (Table 6). However, patients with periodontal infection exhibited significantly higher levels of total cholesterol and Lp(a). Concentrations of LDL‐cholesterol and PCSK9 tended to be more elevated in periodontitis patients compared to those of periodontally healthy subjects.

**TABLE 6:** Link between the periodontal status and the levels of plasma metabolic markers.

Metabolic markers	Absence of periodontitis	Presence of periodontitis	*p*
Triglycerides (g/L)	0.99 ± 0.27	1.31 ± 1.03	0.751
Total cholesterol (g/L)	1.60 ± 0.22	1.75 ± 0.32	0.035*
LDL‐cholesterol (g/L)	0.94 ± 0.16	1.06 ± 0.30	0.071
HDL‐cholesterol (g/L)	0.46 ± 0.10	0.46 ± 0.11	0.900
Lipoprotein (a) (mg/dL)	4.89 ± 0.28	7.08 ± 0.66	0.026*
PCSK9 (mg/L)	11.50 ± 0.88	13.62 ± 0.90	0.099
Glycemia (mmol/L)	5.31 ± 0.81	5.91 ± 1.75	0.346
Insulinemia (mUI/L)	26.11 ± 13.28	29.12 ± 15.15	0.632

*Note:* Blood samples were obtained from patients with (Presence, *n* = 23) or without (Absence, *n* = 13) periodontitis, noting that samples were unavailable for 3 patients without periodontal infection. Circulating levels of glucose, triglycerides, total cholesterol and HDL‐cholesterol were determined by using colorimetric assays, and LDL‐cholesterol levels were calculated according to Friedewald formula. Circulating levels of Lipoprotein (a), PCSK9 and insulin were evaluated by ELISA. Data are expressed as mean ± standard deviation. *p* Values refer to comparisons between the two groups using Mann–Whitney test.

* *p* < 0.05.

### 3.7 Link Between Periodontitis and the Production of Adipokines in the Adipose Tissue

To evaluate the relationship between periodontitis and the secretory function of the visceral adipose tissue, epiploon samples were collected and levels of adipokines measured (Table 7). No significant difference in the levels of IL‐6, MCP‐1, IL‐8, leptin, resistin, and adiponectin was depicted between patients with and without periodontitis.

**TABLE 7:** Link between the periodontal status and the levels of adipokines in the visceral adipose tissue.

Parameters	Absence of periodontitis	Presence of periodontitis	*p*
IL‐6	54.95 ± 55.17	53.44 ± 65.51	0.793
MCP‐1	113.74 ± 99.97	90.27 ± 109.73	0.344
IL‐8	20.95 ± 16.47	24.32 ± 20.57	0.914
Leptin	79.76 ± 32.23	103.49 ± 45.48	0.107
Resistin	26.61 ± 29.70	103.50 ± 273.40	0.963
Adiponectin	3.84 ± 1.08	4.12 ± 1.62	0.769

*Note:* Epiploon adipose tissue samples were obtained from patients with (Presence, *n* = 23) or without (Absence, *n* = 14) periodontitis, noting that samples were unavailable for 2 patients without periodontal infection. Levels of IL‐6, MCP‐1, IL‐8, leptin, resistin, and adiponectin were evaluated by ELISA (pg/mg proteins). Data are expressed as mean ± standard deviation. *p* Values refer to comparisons between the two groups using Mann–Whitney test.

### 3.8 Link Between Periodontitis and Bacterial DNA Presence in the Adipose Tissue

**FIGURE 3:**
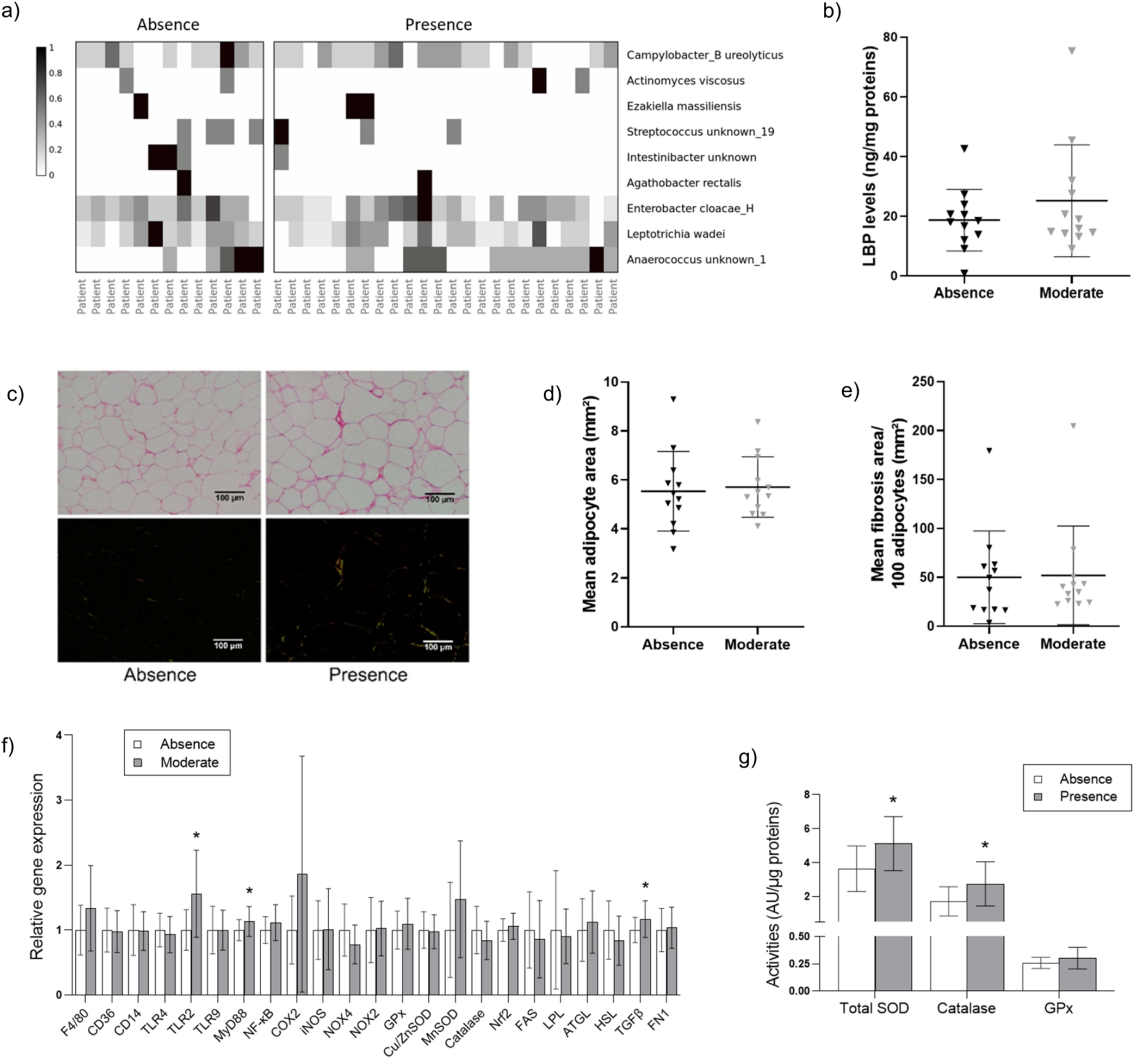
Link between the periodontal status and the inflammatory and metabolic markers in the visceral adipose tissue. Epiploon adipose tissue was obtained from patients with (Presence, *n* = 23) or without (Absence, *n* = 14) periodontitis, noting that samples were unavailable for 2 patients without periodontal infection. DNA extraction from adipose tissue was carried out in a sterile environment then metagenomic analysis was performed. After bioinformatic control for contaminants, 9 bacteria were identified (a). Subsequently, 12 patients without periodontitis (Absence) and 12 patients with moderate periodontitis (Moderate), matched for gender and age were selected. Levels of LBP were measured by ELISA (b). Picrosirius Red staining was performed on 4 μm sections of formalin‐fixed and paraffin‐embedded samples. Representative photographs of Picrosirius Red staining were taken under white light (above) and polarized light (below) (scale: 100 μm) (c). Adipocyte surface area (d) and fibrosis area were quantified (e). The relative expression of genes encoding inflammatory and metabolic markers was evaluated by RT‐qPCR (f). Activities of antioxidant enzymes, including total SOD, catalase, and GPx were determined by colorimetric assay (g). Data are expressed as mean ± standard deviation. Wilcoxon rank‐sum test was performed. *p < 0.05 as compared to Absence of periodontitis.

To assess the presence of bacterial DNA on the visceral adipose tissue, DNA from epiploon samples was extracted, and metagenomic analysis performed. Data indicate that DNA from 9 bacteria was detected in visceral adipose samples (Figure 3a). Nevertheless, none of the detected bacteria led to distinguish patients with and without periodontitis, suggesting that the presence of bacterial DNA in the adipose tissue may not be exclusively attributed to periodontitis. Metagenomic analysis led to the identification of DNA from the genus *Streptococcus*, *Intestinibacter*, and *Anaerococcus* of unknown species. Additionally, DNA from specific bacterial species, including *Campylobacter ureolyticus* , *Actinomyces viscosus* , *Ezakiella massiliensis*, *Agathobacter rectalis*, *Enterobacter cloacae,* and *Leptotrichia wadei* were detected. Most of these bacteria are commonly associated with the intestinal microbiota, suggesting the potential contribution of gut microbiota translocation to visceral adipose tissue colonization. Interestingly, the detection of *Actinomyces viscosus* and *Leptotrichia wadei* , both well‐documented oral cavity bacteria, raises the possibility of bacterial translocation from the oral cavity to the visceral adipose tissue. Although these findings do not demonstrate the presence of periodontal pathogens in this tissue, they are consistent with the possibility that bacteria or bacterial components originating from the oral microbiota may reach the visceral adipose tissue.

### 3.9 Link Between Periodontitis and the Adipose Tissue Inflammatory and Metabolic Profile

To evaluate the link between periodontal infection and the adipose tissue structure and functionality, we measured adipocyte size, fibrosis rate, the production of inflammatory and metabolic markers, as well as LBP levels in the epiploon samples. Data show that LBP levels did not differ significantly between patients with and without periodontitis (Figure 3b). No significant difference in adipocyte size or fibrosis area was depicted between the two groups (Figure 3c–e). The presence of a periodontal infection was not associated with a change in the expression of genes encoding CD14 and TLR4, which are known to mediate LPS signaling by facilitating their transport to the cell surface via LBP (Figure 3f). Furthermore, the expression of genes coding for key immune and inflammatory markers, including NF‐κB, F4/80, CD36, TLR9, COX‐2, and iNOS remained unchanged between groups. Interestingly, we found a significant up‐regulation of *TLR2* gene expression in patients with periodontitis. Concomitantly, a significant increase in the expression of the gene encoding MyD88 was found, keeping in mind that MyD88 is a critical adaptor protein in the TLR/NF‐κB signaling pathway. Moreover, patients with periodontitis exhibited a significant increase in mRNA levels of TGFβ, known to be involved in immune response and fibrosis development. No change was found for the expression of the gene coding for FN1, a key extracellular matrix protein. Moreover, data indicate no change in the expression rate of the genes encoding enzymes involved in lipid metabolism, such as FAS, LPL, ATGL, and HSL. In parallel, the expression of genes encoding ROS‐producing enzymes (NOX2 and NOX4), antioxidant enzymes (GPx, Cu/ZnSOD, MnSOD, catalase), and the redox‐sensitive transcription factor Nrf2 was not different between patients with and without periodontitis. Importantly, a significant increase in total SOD and catalase activities was detected in the adipose tissue of periodontitis patients (Figure 3g), suggesting an adaptive antioxidant response against oxidative stress in the visceral adipose tissue during a periodontal infection.

### 3.10 Correlation Between Periodontitis Parameters, Inflammatory Status, and Metabolic Markers

**FIGURE 4:**
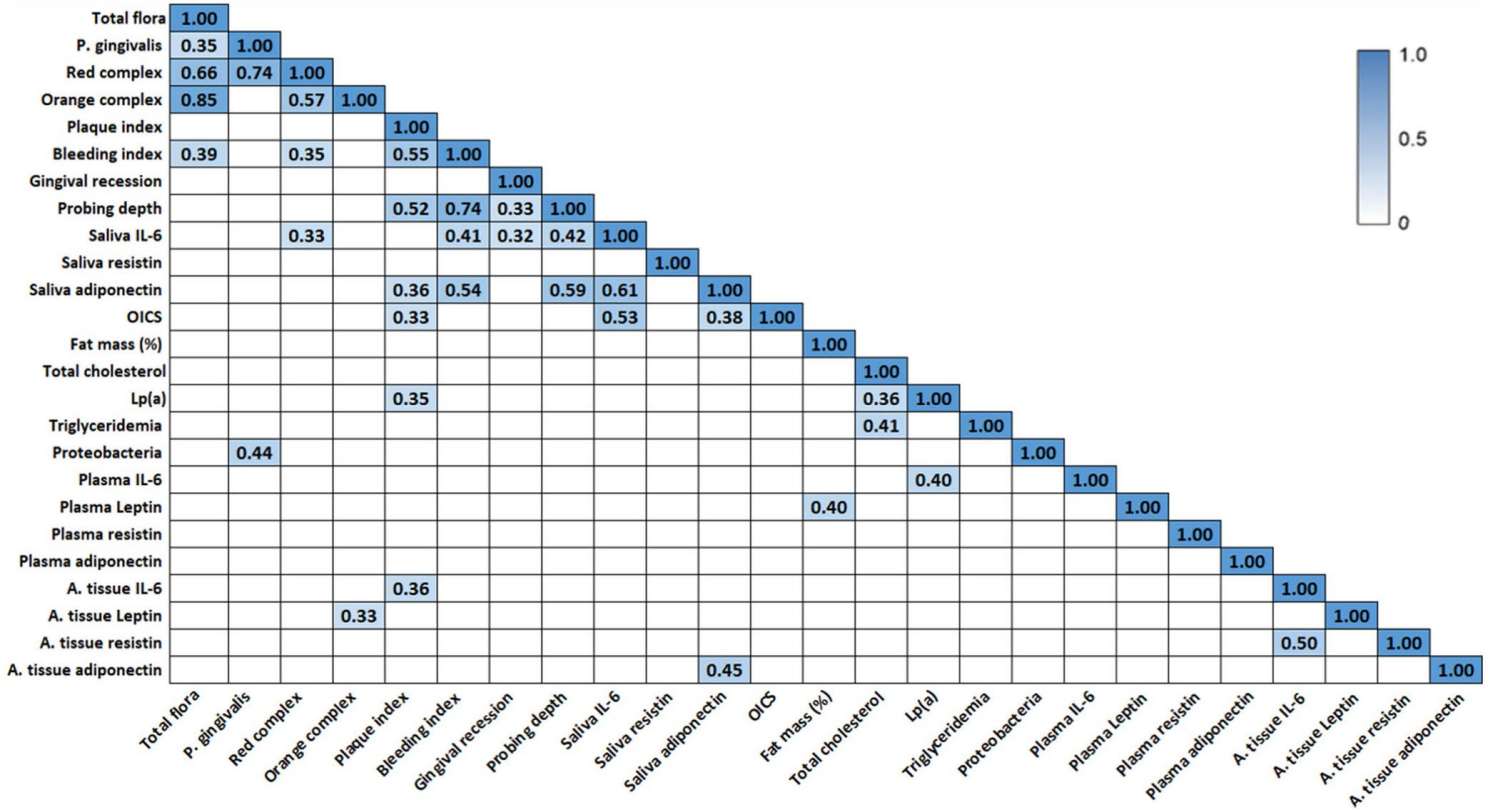
Correlation coefficients between periodontitis‐related parameters, inflammatory status, and metabolic markers. Heat map of the coefficients of correlation between clinical parameters of periodontal health, periodontal bacteria count and selected biological markers. Correlation analyses were performed using Spearman's rank correlation test for highlighting potential relationships between variables. Only significant correlations are reported (*p* < 0.05). Red complex: sum of *Porphyromonas gingivalis* , *Tannerella forsythia* , *Treponema denticola* count. Orange complex: sum of *Prevotella intermedia* , *Peptostreptococcus micros,* and *Fusobacterium nucleatum* . A. tissue, adipose tissue; OICS, oral inflammatory composite score.

Spearman's correlation tests were performed to explore potential associations between periodontal status indicators and inflammatory and metabolic markers. The heat map analysis (Figure 4) highlights significant positive correlations between periodontal health parameters, oral or gut microbiota composition, inflammatory status, and metabolic profile. Indeed, total oral bacteria count correlated with both the orange and red bacterial complexes, as well as with *P. gingivalis* count. A significant correlation between the red and orange bacterial complexes was also observed, in line with their role in periodontal disease progression. Clinical indicators of periodontitis, such as probing depth were positively associated with plaque index, bleeding index, and gingival recession. The total oral bacteria count and the red complex were correlated to the bleeding index, supporting a link between bacterial colonization and periodontal tissue inflammation. Beyond local oral health, several significant associations were identified between periodontal infection and systemic inflammatory and metabolic markers. Circulating concentrations of Lp(a) were positively correlated with those of total cholesterol and IL‐6, and with plaque index. Furthermore, salivary IL‐6 and adiponectin levels were positively correlated with all four periodontal health indicators, and with the red complex, suggesting a relationship between systemic inflammatory response and periodontal infection. Interestingly, the relative abundance of proteobacteria in the gut microbiota was found to correlate specifically with *P. gingivalis* count from periodontal pocket fluid. Regarding the metabolic parameters, leptinemia and fat mass exhibited significant correlations, in line with the ability of adipose tissue to secrete leptin in response to fat storage. Importantly, levels of IL‐6 from the adipose tissue were positively correlated with plaque index, while those of leptin were associated with the presence of orange complex bacteria in periodontal pockets. This suggests an influence of periodontal infection on the adipose tissue inflammatory and metabolic markers. In parallel, significant positive correlations were observed between adipose tissue and salivary adiponectin levels, as well as between adipose tissue resistin and IL‐6 levels, further supporting the systemic impact of periodontitis. Overall, these findings suggest potential interactions between periodontal infection and inflammatory and metabolic parameters at both systemic and adipose tissue levels. Nevertheless, these results should be interpreted as exploratory and will require confirmation in larger studies.

## 4 Discussion

This study evaluated the links between periodontitis markers, the bacterial composition of the gut microbiota, and the inflammatory and metabolic status of patients suffering from severe obesity. Results show that the presence of a periodontal infection correlated with qualitative and quantitative deregulation of the oral microbiota, degradation of tooth‐supporting tissues, and increased levels of salivary pro‐inflammatory markers. These changes were associated with an alteration of the gut microbiota composition and bacterial functional profile, as well as changes in the systemic lipid profile of patients. Furthermore, data indicate that the visceral adipose tissue of patients with periodontitis is marked by the activation of mediators related to inflammation and oxidative stress.

We enrolled 39 patients with grade II or III obesity and reported a periodontitis frequency of 59%, further supporting the strong association between obesity and periodontal disease [6, 47]. A similar frequency was observed in a study including 79 patients with a BMI of 44.6 ± 7.2 kg/m^2^ [48]. The progression of periodontitis is closely linked to the growth of the bacterial biofilm on dental plaque, highlighted in our study by the 10‐fold increase in total oral bacteria count in patients with periodontitis. Among the key bacterial species involved in periodontitis, we assessed the presence and quantified 9 periodontopathogens clinically sought for diagnosing and managing periodontal diseases, namely *P. gingivalis* , *T. forsythia,* and *T. denticola* from the Socransky red complex [8]; *P. intermedia* , *P. micros,* and *F. nucleatum* from the orange complex; and *C. rectus* , *E. corrodens,* and *A. actinomycetemcomitans* . Results show a significant enrichment of red and orange complex bacteria, as well as *C. rectus* , an orange complex‐associated species in patients with periodontitis. In line with our results, sequencing data from previous studies have reported an increased abundance of red and orange complex bacteria during periodontitis [9, 49]. However, in terms of detection rate, a statistical significance was observed only for *P. intermedia* , while *P. micros* and *F. nucleatum* were detected in all patients, regardless of periodontal status. These results support the current concept that periodontitis is driven by a microbial shift toward a dysbiotic microbiota, characterized by changes in bacteria abundance, rather than by colonization by new bacterial species [50, 51]. Red complex bacteria can be present in healthy individuals as a minor fraction of the subgingival microbiota [52, 53], but their abundance increases dramatically with the development of periodontitis [9, 49, 51, 53]. When inflammation progresses, the local microenvironment enriched in tissue breakdown products and plasma proteins, such as hemoglobin, favors the growth of gram‐negative bacteria [54]. Notably, hemoglobin serves as a substrate for proteolytic bacteria like *P. gingivalis* [55]. Consequently, red complex bacteria are well established to be linked to bleeding or probing and increased pocket depth [8, 9, 49]. In agreement with these observations, our data indicate that patients with periodontitis presented worsened clinical parameters, including increased probing depth and clinical attachment level as well as higher bleeding and gingival recession indices, which were doubled and tripled, respectively. Interestingly, the present study identifies *A. actinomycetemcomitans* , a pathogen known to be involved in aggressive periodontitis [56], as a discriminant bacterium for periodontal status in patients with severe obesity.

During periodontitis, the production of various cytokines and chemokines is induced to recruit innate immune cells [57]. Our data show an increase in the salivary concentrations of IL‐6 and adiponectin in patients with periodontal infection, both of which correlated positively with clinical parameters of periodontal health. On the one hand, IL‐6, produced by many cell types in response to bacterial stimuli, is considered a biomarker for the progression of chronic periodontitis [58]. Kawamoto et al. [59] reported higher salivary IL‐6 levels in periodontitis patients compared to healthy individuals, which correlated with increased abundance of red and orange complex bacteria. A similar correlation was observed in the present study. These results are consistent with in vitro experiments indicating that IL‐6 is secreted by macrophages, or human gingival and periodontal cells following exposure to *P. gingivalis* [60], *T. forsythia* [61], *F. nucleatum* [62], and *P. micros* [63] whole bacteria or components. On the other hand, adiponectin, an anti‐inflammatory adipokine originally believed to be exclusively secreted by adipocytes, has also been found in saliva [64] and secreted by salivary gland epithelial cells [65]. However, its precise function in the oral environment remains a topic of debate. Riis et al. [66] found a positive association between oral health, adiponectin salivary level, and markers of oral inflammatory response (IL‐1β, IL‐6, IL‐8, TNFα, and MMP‐8). The calculated Oral Inflammatory Composite Score in our study confirmed this relationship. Of note, the correlation we found between adiponectin salivary level and the bleeding index may suggest that the more elevated salivary level of adiponectin during periodontitis could result from plasma protein leakage [67]. In the study from Riis et al. [66], results did not report significant differences in adiponectin salivary levels between individuals with and without gingival bleeding, but showed a positive association with transferrin salivary levels.

One mechanism linking periodontal infection and systemic complications involves the deregulation of gut microbiota by chronic swallowing of periodontal pathobionts and salivary cytokines produced in the oral cavity. Our data indicate no significant difference in the gut microbiota alpha diversity between patients with and without periodontitis. This could be related to the presence of a gut microbiome already dysbiotic in patients suffering from obesity [2]. However, we found an increase in the relative abundance of the fecal Proteobacteria phylum in patients with periodontitis. Accordingly, Lourenço et al. [68] reported an increase in the relative abundance of Firmicutes, Proteobacteria, Verrucomicrobia, and Euryarchaeota phyla in stool samples collected from individuals with a BMI < 30 kg/m^2^ and a chronic periodontal disease. Proteobacteria constitute one of the most abundant phyla in the human gut microbiota, and their abundance has been associated with metabolic disorders such as obesity and type 2 diabetes, as well as cardiovascular diseases [69, 70]. As gram‐negative bacteria, Proteobacteria produce LPS. In individuals with severe obesity, characterized by increased intestinal permeability, the elevated presence of LPS‐producing bacteria may contribute to endotoxemia, leading to chronic inflammation and associated complications [5, 69, 71]. Interestingly, our data suggest a positive association between the relative abundance of Proteobacteria from the gut microbiota and *P. gingivalis* abundance in the oral cavity, highlighting a potential impact of this pathogen on gut microbiota communities. Arimatsu et al. [29] provided evidence for gut microbiota alterations following oral administration of *P. gingivalis* in mice. These findings are also in line with data from in vivo studies reporting varying degrees of periodontal bacteria's impact on gut microbiota composition [30, 72]. Of note, oral administration of *P. gingivalis* has been shown to increase blood endotoxin levels, to downregulate the expression of tight junction proteins in the ileum, and to induce insulin resistance in mice [29].

At the species levels, our metagenomic analysis of the gut microbiota composition shows an enrichment of several Firmicutes lineages, including *Streptococcus*, *Faecalibacterium*, and *Eubacterium*, as well as *Collinsella* lineages from the Actinobacteria phylum. Among these species, *Faecalibacterium* and *Eubacterium* are known to produce short‐chain fatty acids (SCFAs) such as butyrate from dietary fibers [73, 74]. This finding aligns with our observation of an increased potential of the gut microbiota to degrade complex carbohydrates in patients with periodontitis. Moreover, we found an increased potential of the gut microbiota for the catabolism of both essential and non‐essential amino acids in patients with periodontal infection. It will be interesting to evaluate the plasma concentrations of SCFAs or essential amino acids in order to provide insights into their absorption rate during periodontitis.

Bao et al. [28] previously reported a higher proportion of saliva‐derived bacteria in the fecal microbiota of patients with severe periodontitis (5.88%) compared to those without periodontal infection (0.6%). Similarly, here we found an enrichment of *Streptococcus vestibularis* , a common inhabitant of the mouth vestibule [75], and *Collinsella aerofaciens* , frequently isolated on the tongue of individuals suffering from oral malodor [76]. Purohit et al. [77] demonstrated that supplementation with *C. aerofaciens* in mice fed a high‐fat diet resulted in increased circulating concentration of ethanol, as well as elevated levels of triglycerides and inflammatory markers in the liver, providing insights into the higher abundance of this species in obese and NASH patients. This observation is in agreement with our results showing an increased potential of the gut microbiota for ethanol production in periodontitis patients. Moreover, the genus *Collinsella* has been positively correlated with serum cholesterol level [78, 79]. Although no significant difference in hepatic steatosis frequency was observed in the present study, these findings highlight the potential risk of developing liver diseases, known to be associated with periodontitis [80]. Interestingly, Baima et al. [81] demonstrated that periodontal treatment reduces the salivary carriage of periodontal pathogens and leads to significant changes in gut microbiota, shifting toward a profile more similar to that of healthy individuals. To our knowledge, the present study provides the first evidence, within the same population of patients with severe obesity, that periodontitis is associated with taxonomic and functional profile of the gut microbiota.

During periodontitis, periodontal pockets with exposed capillaries can serve as an entry for periodontal bacteria and their products into the bloodstream, triggering a systemic inflammatory response in the host. Our data did not show any significant differences in plasma and adipose tissue levels of LBP, a well‐established marker of endotoxemia. This could reflect pre‐existing endotoxemia associated with severe obesity, resulting from compromised oral and gut barrier integrity [4, 82]. Interestingly, while no significant difference was observed concerning the saliva, plasma levels of anti‐ *P. gingivalis* IgG were significantly higher in patients with periodontitis, reflecting the establishment of a systemic immune response against this key periodontopathogen. Given that other periodontal bacteria can elicit an immune response, further exploration of IgG response against additional bacterial species would be relevant.

Recent technological advancements help in the identification of tissue‐specific bacterial DNA signatures [83]. In the adipose tissue, these bacterial signatures may vary according to the host's metabolic profile and potentially contribute to inflammation and insulin resistance [84, 85, 86]. We aimed to detect DNA of periodontal bacteria in the visceral adipose tissue of patients with periodontitis, using metagenomic analysis, and validated the detection of DNA from 9 different bacteria. Nevertheless, we did not depict differences in bacterial DNA prevalence between patients with and without periodontitis. Notably, all identified bacteria were anaerobe or facultative anaerobe species. The detected bacteria belonged to the phyla previously described by Sun et al. [86], including Proteobacteria, Firmicutes, Actinobacteriota and Fusobacteriota. Among them, *Agathobacter rectalis* and *Enterobacter cloacae* are known gut commensals [87, 88], while *Campylobacter ureolyticus* has been reported as a gastrointestinal pathogen [89]. *Agathobacter rectalis*, a butyrate‐producing bacterium, has been shown to alleviate microglia‐mediated neuroinflammation via its metabolite butyrate [88]. Interestingly, our analysis identified two bacterial species of oral origin, namely *Leptotrichia wadei* and *Actinomyces viscosus* . *Leptotrichia wadei* is a bacterium commonly found in the oral cavity and isolated from saliva [90]. *Actinomyces viscosus* is a gram‐positive bacterium that colonizes the oral cavity of most adults and is frequently found in dental plaque and buccal mucosa samples [91]. Both bacteria may have originated either from the oral cavity or from the intestinal microbiota through swallowed saliva. Of note, Hsiao et al. [92] reported *A. viscosus* bacteremia in patients with poor oral hygiene and chronic periodontitis. In addition, bacteria from the *Anaerococcus* genus are commonly found in the oral, upper respiratory, and intestinal tracts. *Ezakiella massiliensis*, originally isolated from the female genital tract [93], was identified only in samples from females in our study, suggesting the possibility of an alternative source of bacteria translocation. To our knowledge, this is the first study to specifically identify bacteria originating from the oral cavity, particularly from dental plaque, in the visceral adipose tissue. However, our results are limited by the low bacteria biomass in the adipose tissue samples, which prevented us from establishing a definitive bacterial signature in the adipose tissue.

Growing literature data demonstrate that patients with periodontitis exhibit increased levels of pro‐inflammatory markers such as CRP, IL‐6, and TNFα [12, 94, 95]. In contrast, in our study, we did not observe a significant association between periodontitis and inflammatory markers in plasma and adipose tissue. This may be due to the pre‐existing low‐grade inflammatory state in patients with severe obesity, which could mask the inflammatory response during periodontal infection. In parallel, other inflammatory pathways not explored in our study may be involved. In particular, the IL‐17/Th17 axis has emerged as a key mediator linking periodontitis to systemic inflammatory and metabolic disorders [96]. A recent experimental study has provided evidence that IL‐17‐driven immune response, notably in response to *P. gingivalis* , can contribute to periodontal inflammation, glucose intolerance, and adipose tissue dysfunction [97]. This pathway may therefore represent an alternative mechanism underlying the systemic effects of periodontal infection in obesity. Moreover, the development of an immune response against *P. gingivalis* may have also contributed. Blasco‐Baque et al. [98] reported that the development of antibodies against *P. gingivalis* conferred protection against periodontitis‐induced metabolic deregulations in mice. Regarding the visceral adipose tissue, although no significant difference was observed concerning its secretory function, our study provides subtle evidence for alterations in markers related to inflammatory and metabolic status in patients suffering from obesity and periodontitis. More particularly, the expression of genes encoding TLR2 and MyD88 was significantly higher in the periodontitis group. Interestingly, we previously demonstrated that *P. gingivalis* LPS induces inflammation through the activation of TLR2 and downstream NF‐κB signaling via MyD88 adaptor [34]. In mouse models administrated with *P. gingivalis* , an enhancement of IL‐6/JAK/STAT3 signaling has been observed in the adipose tissue, along with increased TLR2 production [99]. Here, to complete the quantification of TLR2 and MyD88 mRNA levels, it will be of interest to use complementary approaches based on the analysis of their protein levels to provide functional data regarding the signaling pathways possibly involved. Such pathways are known to act as a central hub, potentially leading to further activation of other signaling cascades. For instance, our findings indicate an increase in TGF‐β gene expression, despite the absence of differences in adipocyte size and fibrosis area between patients with and without periodontitis. This increase in TGF‐β gene expression in the visceral adipose tissue of patients suffering from obesity without a corresponding increase in the area of fibrosis may reflect an early stage of tissue remodeling that has not yet resulted in measurable collagen deposition. This is particularly relevant in the context of severe obesity, where baseline tissue remodeling may already be present. Beyond its well‐established role in fibrogenesis, TGF‐β is a pleiotropic cytokine involved in immune regulation and tissue homeostasis [100]. Therefore, its upregulation may indicate early immunomodulatory or remodeling processes that precede detectable collagen deposition, as fibrosis is a dynamic and progressive process [101].

Periodontitis is well known to be associated with oxidative stress [102]. Oxidative stress is caused by an imbalance between the overproduction of highly reactive molecular species, such as ROS, and the endogenous antioxidant defense capacity [103]. During periodontitis, neutrophils, which contribute to the first line of defense against bacterial infection, exhibit a hyperactive phenotype and produce increased amounts of ROS [104]. Our data demonstrate a significant increase in total SOD and catalase enzymatic activities in the visceral adipose tissue collected from patients with periodontitis. Interestingly, this increase in enzymatic activity occurred without changes in SOD and catalase mRNA levels, suggesting a regulation beyond the transcriptional level. In the context of obesity‐associated oxidative stress, antioxidant enzyme gene expression may already be elevated, limiting further transcriptional induction [105]. Periodontitis may therefore act as an additional stimulus, promoting the functional activation of pre‐existing enzyme pools through post‐transcriptional or post‐translational mechanisms, including changes in protein stability, cofactor availability, and redox‐dependent modifications [106]. Both SOD and catalase are antioxidant enzymes that protect cells against ROS by scavenging superoxide radical and hydrogen peroxide, respectively. The relationship between periodontal status and antioxidant enzyme activity remains controversial in the literature. Most studies report a decrease in antioxidant enzyme activity during periodontitis [107]. In contrast, Panjamurthy et al. [108] found significantly higher enzymatic activities of SOD, catalase, and GPx in plasma, erythrocytes, and gingival tissues of periodontitis patients compared to healthy controls, supporting our findings. Such an activation of the endogenous antioxidant defense system may reflect increased oxidative stress within the affected tissues in patients with periodontitis. In the adipose tissue, elevated ROS levels are often linked to the activation of immune cells (e.g., macrophages and neutrophils) but may also arise from direct oxidative stress on adipocytes. Consistently, we previously reported that *P. gingivalis* LPS induces oxidative stress in adipocytes through the activation of TLR2 and NADPH oxidases known as ROS‐producing enzymes [34]. Within the adipose tissue, oxidative stress can disrupt homeostasis and contribute to insulin resistance [109]. Accordingly, periodontitis is associated with increased oxidative stress and compromised glycemic control in patients suffering from type 2 diabetes [110]. Interestingly, *A. viscosus* , which we detected here in the visceral adipose tissue, has been shown to increase total ROS release by peripheral blood neutrophils [111].

Consistent with previous studies highlighting the influence of periodontal infection on lipid metabolism, our findings show significantly higher circulating levels of total cholesterol, Lp(a), and a tendency toward elevated LDL‐cholesterol (*p* = 0.071) in patients with periodontitis. Furthermore, our data indicate that only patients with periodontitis exhibited dyslipidemia as a comorbidity factor and received a lipid‐lowering treatment, compared to patients without periodontal infection. Of note, in the periodontitis group, only a minority of patients exhibited dyslipidemia (3 out of 23 patients) and received a lipid‐lowering treatment (4 out of 23 patients), suggesting that this imbalance is unlikely to fully account for the observed differences. Consistently, the distribution of lipid parameters suggests a global shift toward higher values in patients with periodontitis, rather than an effect driven by a small subset of individuals. Several clinical studies reported a positive correlation between periodontitis and increased serum levels of total cholesterol and LDL‐cholesterol [13, 14, 112]. Basdorf et al. [113] reported that periodontitis is associated not only with elevated total cholesterol, LDL‐cholesterol, and triglycerides levels, but also with changes in lipoprotein subfractions. More particularly, higher levels of cholesterol‐ and triglyceride‐enriched Apo‐B‐containing lipoproteins, such as small dense LDL (sdLDL), very‐low‐density lipoprotein (VLDL), and intermediate‐density lipoprotein (IDL) particles, as well as alterations in HDL particle composition, have been observed. Investigating these lipoprotein subfractions in our study could provide deeper insight into the lipid profile disruptions associated with periodontal disease. Animal models have provided mechanistic insights into this relationship. In mice, intraperitoneal injection of *P. gingivalis* led to an elevation of serum LDL‐cholesterol levels and an increase in circulating concentrations of PCSK9, a protein known to promote LDL receptor degradation [114]. Our study tended to demonstrate the same association concerning more elevated levels of PSCK9 (*p* = 0.099) in patients with periodontitis. In line with these findings, chronic oral infection with *P. gingivalis* in mice was associated with a tendency toward reduced production of hepatic LDL receptors in the liver, along with increased total cholesterol and LDL‐cholesterol levels [115]. Importantly, periodontal therapy has been shown to reduce serum triglycerides, total cholesterol, and LDL‐cholesterol levels [116, 117, 118], reinforcing the concept that treating periodontal disease may have a beneficial effect on lipid profile. These alterations in lipid metabolism may have significant implications for cardiovascular (CV) risk. In parallel, our results show increased circulating levels of Lp(a) in patients with periodontitis. Lp(a) is an atherogenic lipoprotein and a recognized biomarker of CV risk. Its concentrations are primarily determined by polymorphisms in the *apo(a)* locus but can be influenced by non‐genetic factors [119]. During bacterial infection, cytokines produced by the host immune response may contribute to elevated Lp(a) levels [120]. Collectively, the findings suggest an increased risk of CV events in patients with periodontitis, corroborating existing literature data [121]. Interestingly, in the present study, altered lipid profiles were still observed despite the presence of lipid‐lowering therapy in the group of patients with periodontitis. This may indicate a more persistent metabolic dysregulation in patients with a periodontal infection. Nevertheless, the potential influence of such treatments cannot be excluded and should be taken into account when interpreting these findings.

The present study provides valuable information regarding the links between periodontitis and the alteration of inflammatory and metabolic status in patients suffering from severe obesity. However, limitations could be considered. Firstly, the small sample size may have limited the statistical power to detect significant differences for some markers, as evidenced by several markers that only showed a tendency toward alteration without reaching significance. Secondly, due to the limited sample size, we included all patients with periodontitis in the same group, although they had varying grades of periodontitis (mild, moderate, severe). In our study, this may have introduced variability and potentially diluted specific associations. Moreover, the pre‐existing chronic low‐grade inflammatory state inherent in severe obesity may have masked the well‐established inflammatory burden induced by periodontitis [12, 94, 95]. The lack of a non‐obese control group also limited our ability to isolate the specific contribution of periodontitis to systemic inflammation related to severe obesity. Our study design does not allow to determine whether the metabolic and inflammatory changes observed are driven by the direct impact of periodontal bacteria or an indirect effect mediated by gut microbiota dysbiosis. Understanding the relative contributions of these pathways would require further mechanistic investigations.

To conclude, this study provides new insights into the intricate relationship between periodontitis, inflammatory status, and metabolic alterations in individuals with severe obesity. Notably, our results show for the first time that periodontitis is associated with changes in the gut microbiota composition and bacterial functional profiles in patients suffering from severe obesity. Periodontal infection parameters were linked to systemic markers, particularly those involved in lipid metabolism, suggesting potential implications for the liver and cardiovascular health. Beyond systemic alterations, the presence of periodontal infection is associated with inflammatory alterations and oxidative stress within the visceral adipose tissue. These findings underscore the importance of considering periodontal health in the prevention of obesity‐related complications. Thus, addressing the periodontal status may not only improve oral health but also contribute to better metabolic outcomes and overall well‐being in patients with obesity.

## Author Contributions

F.S., O.M., N.L.M. and M.‐P.G. contributed to the conceptualization. K.T., F.S., V.L., E.D., E.B., R.A., N.L.M. and M.‐P.G. contributed to the methodology. K.T., F.S., J.T., R.A., P.R., N.L.M. and M.‐P.G. contributed to the data acquisition. K.T., V.L., E.D., K.C., O.M., N.L.M. and M.‐P.G. were involved in the data analysis and interpretation, and the writing of the manuscript. All authors have read and agreed to the published version of the manuscript.

## Funding

This research was funded by the CHU de La Réunion (APIDOM‐BACTERIOB), the Institut National de la Santé et de la Recherche Médicale (Inserm‐Programme Transversal Microbiote PTM2) and the University of La Réunion.

## Conflicts of Interest

The authors declare no conflicts of interest.

## Data Availability

The original contributions presented in this study are included in the article. Further inquiries can be directed to the corresponding author.

## References

[1] World Health Organization , Global Report on Diabetes (World Health Organization, 2016) 83 p.

[2] J. Breton , M. Galmiche , and P. Déchelotte , “Dysbiotic Gut Bacteria in Obesity: An Overview of the Metabolic Mechanisms and Therapeutic Perspectives of Next‐Generation Probiotics,” Microorganisms 10, no. 2 (2022): 452, 10.3390/microorganisms10020452. 35208906 PMC8877435

[3] C. Pedersen , U. Z. Ijaz , E. Gallagher , et al., “Fecal Enterobacteriales Enrichment Is Associated With Increased In Vivo Intestinal Permeability in Humans,” Physiological Reports 6, no. 7 (2018): e13649, 10.14814/phy2.13649. 29611319 PMC5880877

[4] P. D. Cani , R. Bibiloni , C. Knauf , et al., “Changes in Gut Microbiota Control Metabolic Endotoxemia‐Induced Inflammation in High‐Fat Diet‐Induced Obesity and Diabetes in Mice,” Diabetes 57, no. 6 (2008): 1470–1481, 10.2337/db07-1403. 18305141

[5] P. D. Cani , J. Amar , M. A. Iglesias , et al., “Metabolic Endotoxemia Initiates Obesity and Insulin Resistance,” Diabetes 56, no. 7 (2007): 1761–1772, 10.2337/db06-1491. 17456850

[6] T. Saito , Y. Shimazaki , T. Koga , M. Tsuzuki , and A. Ohshima , “Relationship Between Upper Body Obesity and Periodontitis,” Journal of Dental Research 80, no. 7 (2001): 1631–1636, 10.1177/00220345010800070701. 11597023

[7] B. W. Chaffee and S. J. Weston , “Association Between Chronic Periodontal Disease and Obesity: A Systematic Review and Meta‐Analysis,” Journal of Periodontology 81, no. 12 (2010): 1708–1724, 10.1902/jop.2010.100321. 20722533 PMC3187554

[8] S. S. Socransky , A. D. Haffajee , M. A. Cugini , C. Smith , and R. L. Kent , “Microbial Complexes in Subgingival Plaque,” Journal of Clinical Periodontology 25, no. 2 (1998): 134–144, 10.1111/j.1600-051X.1998.tb02419.x. 9495612

[9] R. P. Teles , D. Sakellari , F. R. F. Teles , et al., “Relationships Among Gingival Crevicular Fluid Biomarkers, Clinical Parameters of Periodontal Disease, and the Subgingival Microbiota,” Journal of Periodontology 81, no. 1 (2010): 89–98, 10.1902/jop.2009.090397. 20059421 PMC2805280

[10] J. Meyle and I. Chapple , “Molecular Aspects of the Pathogenesis of Periodontitis,” Periodontology 2000 69, no. 1 (2015): 7–17, 10.1111/prd.12104. 26252398

[11] G. Hajishengallis and T. Chavakis , “Local and Systemic Mechanisms Linking Periodontal Disease and Inflammatory Comorbidities,” Nature Reviews Immunology 21 (2021): 1–15, 10.1038/s41577-020-00488-6. PMC7841384 33510490

[12] R. T. Demmer , A. Squillaro , P. N. Papapanou , et al., “Periodontal Infection, Systemic Inflammation, and Insulin Resistance,” Diabetes Care 35, no. 11 (2012): 2235–2242, 10.2337/dc12-0072. 22837370 PMC3476901

[13] W. Lösche , F. Karapetow , A. Pohl , C. Pohl , and T. Kocher , “Plasma Lipid and Blood Glucose Levels in Patients With Destructive Periodontal Disease,” Journal of Clinical Periodontology 27, no. 8 (2000): 537–541, 10.1034/j.1600-051x.2000.027008537.x. 10959778

[14] S. A. Moghaddam , S. Abbasi , E. S. Moghaddam , and A. A. Moghaddam , “Triglyceride and Cholesterol Levels in Patients With Chronic Periodontitis,” Health Scope 4, no. 2 (2015): 2, 10.17795/jhealthscope-19928.

[15] F. D'Aiuto , N. Gkranias , D. Bhowruth , et al., “Systemic Effects of Periodontitis Treatment in Patients With Type 2 Diabetes: A 12 Month, Single‐Centre, Investigator‐Masked, Randomised Trial,” Lancet Diabetes and Endocrinology 6, no. 12 (2018): 954–965, 10.1016/S2213-8587(18)30038-X. 30472992

[16] C. G. Daly , D. H. Mitchell , J. E. Highfield , D. E. Grossberg , and D. Stewart , “Bacteremia due to Periodontal Probing: A Clinical and Microbiological Investigation,” Journal of Periodontology 72, no. 2 (2001): 210–214, 10.1902/jop.2001.72.2.210. 11288795

[17] A. C. R. T. Horliana , L. Chambrone , A. M. Foz , et al., “Dissemination of Periodontal Pathogens in the Bloodstream After Periodontal Procedures: A Systematic Review,” PLoS One 9, no. 5 (2014): e98271, 10.1371/journal.pone.0098271. 24870125 PMC4037200

[18] S. O. Geerts , M. Nys , P. De Mol , et al., “Systemic Release of Endotoxins Induced by Gentle Mastication: Association With Periodontitis Severity,” Journal of Periodontology 73, no. 1 (2002): 73–78, 10.1902/jop.2002.73.1.73. 11846202

[19] I. Tomás , P. Diz , A. Tobías , C. Scully , and N. Donos , “Periodontal Health Status and Bacteraemia From Daily Oral Activities: Systematic Review/Meta‐Analysis,” Journal of Clinical Periodontology 39, no. 3 (2012): 213–228, 10.1111/j.1600-051X.2011.01784.x. 22092606

[20] M. Stelzel , G. Conrads , S. Pankuweit , et al., “Detection of *Porphyromonas gingivalis* DNA in Aortic Tissue by PCR,” Journal of Periodontology 73, no. 8 (2002): 868–870, 10.1902/jop.2002.73.8.868. 12211495

[21] K. Nakano , H. Nemoto , R. Nomura , et al., “Detection of Oral Bacteria in Cardiovascular Specimens,” Oral Microbiology and Immunology 24, no. 1 (2009): 64–68, 10.1111/j.1399-302X.2008.00479.x. 19121072

[22] S. S. Dominy , C. Lynch , F. Ermini , et al., “ *Porphyromonas gingivalis* in Alzheimer's Disease Brains: Evidence for Disease Causation and Treatment With Small‐Molecule Inhibitors,” Science Advances 5, no. 1 (2019): eaau3333, 10.1126/sciadv.aau3333. 30746447 PMC6357742

[23] V. Ilievski , P. T. Toth , K. Valyi‐Nagy , et al., “Identification of a Periodontal Pathogen and Bihormonal Cells in Pancreatic Islets of Humans and a Mouse Model of Periodontitis,” Scientific Reports 10, no. 1 (2020): 9976, 10.1038/s41598-020-65828-x. 32561770 PMC7305306

[24] S. Delbosc , J. M. Alsac , C. Journe , et al., “ *Porphyromonas gingivalis* Participates in Pathogenesis of Human Abdominal Aortic Aneurysm by Neutrophil Activation. Proof of Concept in Rats,” PLoS One 6, no. 4 (2011): e18679, 10.1371/journal.pone.0018679. 21533243 PMC3076426

[25] A. Freiherr Von Seckendorff , M. S. Nomenjanahary , J. Labreuche , et al., “Periodontitis in Ischemic Stroke: Impact of *Porphyromonas gingivalis* on Thrombus Composition and Ischemic Stroke Outcomes,” Research and Practice in Thrombosis and Haemostasis 8, no. 1 (2024): 102313, 10.1016/j.rpth.2023.102313. 38318152 PMC10840352

[26] L. Winning , C. C. Patterson , K. M. Cullen , et al., “The Association Between Subgingival Periodontal Pathogens and Systemic Inflammation,” Journal of Clinical Periodontology 42, no. 9 (2015): 799–806, 10.1111/jcpe.12450. 26309048

[27] S. P. Humphrey and R. T. Williamson , “A Review of Saliva: Normal Composition, Flow, and Function,” Journal of Prosthetic Dentistry 85, no. 2 (2001): 162–169, 10.1067/mpr.2001.113778. 11208206

[28] J. Bao , L. Li , Y. Zhang , et al., “Periodontitis May Induce Gut Microbiota Dysbiosis via Salivary Microbiota,” International Journal of Oral Science 14, no. 1 (2022): 32, 10.1038/s41368-022-00183-3. 35732628 PMC9217941

[29] K. Arimatsu , H. Yamada , H. Miyazawa , et al., “Oral Pathobiont Induces Systemic Inflammation and Metabolic Changes Associated With Alteration of Gut Microbiota,” Scientific Reports 4, no. 1 (2015): 4828, 10.1038/srep04828. PMC4010932 24797416

[30] T. Kato , K. Yamazaki , M. Nakajima , et al., “Oral Administration of *Porphyromonas gingivalis* Alters the Gut Microbiome and Serum Metabolome,” mSphere 3, no. 5 (2018): e00460‐18, 10.1128/mSphere.00460-18. 30333180 PMC6193602

[31] N. Ouchi , J. L. Parker , J. J. Lugus , and K. Walsh , “Adipokines in Inflammation and Metabolic Disease,” Nature Reviews Immunology 11, no. 2 (2011): 85–97, 10.1038/nri2921. PMC3518031 21252989

[32] Y. S. Lee , P. Li , J. Y. Huh , et al., “Inflammation Is Necessary for Long‐Term but Not Short‐Term High‐Fat Diet–Induced Insulin Resistance,” Diabetes 60, no. 10 (2011): 2474, 10.2337/db11-0194. 21911747 PMC3178297

[33] Y. Huang , J. Zeng , G. Chen , X. Xie , W. Guo , and W. Tian , “Periodontitis Contributes to Adipose Tissue Inflammation Through the NF‐B, JNK and ERK Pathways to Promote Insulin Resistance in a Rat Model,” Microbes and Infection 18, no. 12 (2016): 804–812, 10.1016/j.micinf.2016.08.002. 27565999

[34] F. Le Sage , O. Meilhac , and M. P. Gonthier , “ *Porphyromonas gingivalis* Lipopolysaccharide Induces Pro‐Inflammatory Adipokine Secretion and Oxidative Stress by Regulating Toll‐Like Receptor‐Mediated Signaling Pathways and Redox Enzymes in Adipocytes,” Molecular and Cellular Endocrinology 446 (2017): 102–110, 10.1016/j.mce.2017.02.022. 28216438

[35] P. I. Eke , R. C. Page , L. Wei , G. Thornton‐Evans , and R. J. Genco , “Update of the Case Definitions for Population‐Based Surveillance of Periodontitis,” Journal of Periodontology 83, no. 12 (2012): 1449–1454, 10.1902/jop.2012.110664. 22420873 PMC6005373

[36] P. I. Eke , B. A. Dye , L. Wei , et al., “Update on Prevalence of Periodontitis in Adults in the United States: NHANES 2009 to 2012,” Journal of Periodontology 86, no. 5 (2015): 611–622, 10.1902/jop.2015.140520. 25688694 PMC4460825

[37] H. Löe , “The Gingival Index, the Plaque Index and the Retention Index Systems,” Journal of Periodontology 38, no. 6P2 (1967): 610–616, 10.1902/jop.1967.38.6_part2.610. 5237684

[38] J. Silness and H. Loe , “Periodontal Disease in Pregnancy II. Correlation Between Oral Hygiene and Periodontal Condition,” Acta Odontologica Scandinavica 22 (1964): 121–135, 10.3109/00016356408993968. 14158464

[39] A. Bainor , L. Chang , T. J. McQuade , B. Webb , and J. E. Gestwicki , “Bicinchoninic Acid (BCA) Assay in Low Volume,” Analytical Biochemistry 410, no. 2 (2011): 310–312, 10.1016/j.ab.2010.11.015. 21078286

[40] P. J. Pussinen , T. Vilkuna‐Rautiainen , G. Alfthan , K. Mattila , and S. Asikainen , “Multiserotype Enzyme‐Linked Immunosorbent Assay as a Diagnostic Aid for Periodontitis in Large‐Scale Studies,” Journal of Clinical Microbiology 40, no. 2 (2002): 512–518, 10.1128/JCM.40.2.512-518.2002. 11825965 PMC153358

[41] J. Patche , D. Girard , A. Catan , et al., “Diabetes‐Induced Hepatic Oxidative Stress: A New Pathogenic Role for Glycated Albumin,” Free Radical Biology and Medicine 102 (2017): 133–148, 10.1016/j.freeradbiomed.2016.11.026. 27890722

[42] A. Divoux , J. Tordjman , D. Lacasa , et al., “Fibrosis in Human Adipose Tissue: Composition, Distribution, and Link With Lipid Metabolism and Fat Mass Loss,” Diabetes 59, no. 11 (2010): 2817–2825, 10.2337/db10-0585. 20713683 PMC2963540

[43] K. J. Livak and T. D. Schmittgen , “Analysis of Relative Gene Expression Data Using Real‐Time Quantitative PCR and the 2^−ΔΔCT^ Method,” Methods 25, no. 4 (2001): 402–408, 10.1006/meth.2001.1262. 11846609

[44] R. Alili , E. Belda , P. Le , et al., “Exploring Semi‐Quantitative Metagenomic Studies Using Oxford Nanopore Sequencing: A Computational and Experimental Protocol,” Genes (Basel) 12, no. 10 (2021): 1496, 10.3390/genes12101496. 34680891 PMC8536095

[45] P. J. McMurdie and S. Holmes , “Phyloseq: An R Package for Reproducible Interactive Analysis and Graphics of Microbiome Census Data,” PLoS One 8, no. 4 (2013): e61217, 10.1371/journal.pone.0061217. 23630581 PMC3632530

[46] Y. Darzi , G. Falony , S. Vieira‐Silva , and J. Raes , “Towards Biome‐Specific Analysis of Meta‐Omics Data,” ISME Journal 10, no. 5 (2016): 1025–1028, 10.1038/ismej.2015.188. 26623543 PMC5029225

[47] M. Martinez‐Herrera , J. Silvestre‐Rangil , and F. J. Silvestre , “Association Between Obesity and Periodontal Disease. A Systematic Review of Epidemiological Studies and Controlled Clinical Trials,” Medicina Oral, Patología Oral y Cirugía Bucal 22, no. 6 (2017): e708–e715, 10.4317/medoral.21786. 29053651 PMC5813989

[48] D. Čolak , A. Cmok Kučič , T. Pintar , B. Gašpirc , and R. Gašperšič , “Periodontal and Systemic Health of Morbidly Obese Patients Eligible for Bariatric Surgery: A Cross‐Sectional Study,” BMC Oral Health 22, no. 1 (2022): 174, 10.1186/s12903-022-02207-0. 35562737 PMC9107195

[49] M. E. Kirst , E. C. Li , B. Alfant , et al., “Dysbiosis and Alterations in Predicted Functions of the Subgingival Microbiome in Chronic Periodontitis,” Applied and Environmental Microbiology 81, no. 2 (2015): 783–793, 10.1128/AEM.02712-14. 25398868 PMC4277562

[50] P. M. Bartold and T. E. Van Dyke , “Periodontitis: A Host‐Mediated Disruption of Microbial Homeostasis. Unlearning Learned Concepts,” Periodontology 2000 62, no. 1 (2013): 203–217, 10.1111/j.1600-0757.2012.00450.x. 23574467 PMC3692012

[51] G. Hajishengallis and R. J. Lamont , “Beyond the Red Complex and Into More Complexity: The Polymicrobial Synergy and Dysbiosis (PSD) Model of Periodontal Disease Etiology,” Molecular Oral Microbiology 27, no. 6 (2012): 409–419, 10.1111/j.2041-1014.2012.00663.x. 23134607 PMC3653317

[52] A. L. Griffen , M. R. Becker , S. R. Lyons , M. L. Moeschberger , and E. J. Leys , “Prevalence of Porphyromonas Gingivalis and Periodontal Health Status,” Journal of Clinical Microbiology 36, no. 11 (1998): 3239–3242. 9774572 10.1128/jcm.36.11.3239-3242.1998 PMC105308

[53] G. Mayanagi , T. Sato , H. Shimauchi , and N. Takahashi , “Detection Frequency of Periodontitis‐Associated Bacteria by Polymerase Chain Reaction in Subgingival and Supragingival Plaque of Periodontitis and Healthy Subjects,” Oral Microbiology and Immunology 19, no. 6 (2004): 379–385, 10.1111/j.1399-302x.2004.00172.x. 15491463

[54] A. A. Abdulkareem , F. B. Al‐Taweel , A. J. B. Al‐Sharqi , S. S. Gul , A. Sha , and I. L. C. Chapple , “Current Concepts in the Pathogenesis of Periodontitis: From Symbiosis to Dysbiosis,” Journal of Oral Microbiology 15, no. 1 (2023): 2197779, 10.1080/20002297.2023.2197779. 37025387 PMC10071981

[55] T. Olczak , W. Simpson , X. Liu , and C. A. Genco , “Iron and Heme Utilization in *Porphyromonas gingivalis* ,” FEMS Microbiology Reviews 29, no. 1 (2005): 119–144, 10.1016/j.femsre.2004.09.001. 15652979

[56] B. Henderson , J. M. Ward , and D. Ready , “ *Aggregatibacter* (*Actinobacillus*) *Actinomycetemcomitans*: A Triple A* Periodontopathogen?,” Periodontology 2000 54, no. 1 (2010): 78–105, 10.1111/j.1600-0757.2009.00331.x. 20712635

[57] D. E. Ramadan , N. Hariyani , R. Indrawati , R. D. Ridwan , and I. Diyatri , “Cytokines and Chemokines in Periodontitis,” European Journal of Dentistry 14, no. 3 (2020): 483–495, 10.1055/s-0040-1712718. 32575137 PMC7440949

[58] H. Batool , A. Nadeem , M. Kashif , F. Shahzad , R. Tahir , and N. Afzal , “Salivary Levels of IL‐6 and IL‐17 Could be an Indicator of Disease Severity in Patients With Calculus Associated Chronic Periodontitis,” BioMed Research International 2018 (2018): 8531961, 10.1155/2018/8531961. 29670909 PMC5835283

[59] D. Kawamoto , P. P. L. Amado , E. Albuquerque‐Souza , et al., “Chemokines and Cytokines Profile in Whole Saliva of Patients With Periodontitis,” Cytokine 135 (2020): 155197, 10.1016/j.cyto.2020.155197. 32707521

[60] M. Yee , S. Kim , P. Sethi , N. Düzgüneş , and K. Konopka , “ *Porphyromonas gingivalis* Stimulates IL‐6 and IL‐8 Secretion in GMSM‐K, HSC‐3 and H413 Oral Epithelial Cells,” Anaerobe 28 (2014): 62–67, 10.1016/j.anaerobe.2014.05.011. 24887636

[61] A. Hasebe , A. Yoshimura , T. Into , et al., “Biological Activities of *Bacteroides forsythus* Lipoproteins and Their Possible Pathological Roles in Periodontal Disease,” Infection and Immunity 72, no. 3 (2004): 1318–1325, 10.1128/IAI.72.3.1318-1325.2004. 14977934 PMC356049

[62] W. Kang , X. Ji , X. Zhang , D. Tang , and Q. Feng , “Persistent Exposure to *Fusobacterium nucleatum* Triggers Chemokine/Cytokine Release and Inhibits the Proliferation and Osteogenic Differentiation Capabilities of Human Gingiva‐Derived Mesenchymal Stem Cells,” Frontiers in Cellular and Infection Microbiology 9 (2019): 429, 10.3389/fcimb.2019.00429. 31921705 PMC6927917

[63] S. i. Tanabe , C. Bodet , and D. Grenier , “ *Peptostreptococcus micros* Cell Wall Elicits a Pro‐Inflammatory Response in Human Macrophages,” Journal of Endotoxin Research 13, no. 4 (2007): 219–226, 10.1177/0968051907081869. 17956940

[64] M. Toda , R. Tsukinoki , and K. Morimoto , “Measurement of Salivary Adiponectin Levels,” Acta Diabetologica 44, no. 1 (2007): 20–22, 10.1007/s00592-007-0236-8. 17357881

[65] S. Katsiougiannis , E. K. Kapsogeorgou , M. N. Manoussakis , and F. N. Skopouli , “Salivary Gland Epithelial Cells: A New Source of the Immunoregulatory Hormone Adiponectin,” Arthritis and Rheumatism 54, no. 7 (2006): 2295–2299, 10.1002/art.21944. 16802369

[66] J. L. Riis , C. I. Bryce , T. Ha , et al., “Adiponectin: Serum‐Saliva Associations and Relations With Oral and Systemic Markers of Inflammation,” Peptides 91 (2017): 58–64, 10.1016/j.peptides.2017.03.006. 28363793

[67] Y. M. Henskens , U. van der Velden , E. C. Veerman , and A. V. Nieuw Amerongen , “Protein, Albumin and Cystatin Concentrations in Saliva of Healthy Subjects and of Patients With Gingivitis or Periodontitis,” Journal of Periodontal Research 28, no. 1 (1993): 43–48, 10.1111/j.1600-0765.1993.tb01049.x. 8426281

[68] T. G. B. Lourenςo , S. J. Spencer , E. J. Alm , and A. P. V. Colombo , “Defining the Gut Microbiota in Individuals With Periodontal Diseases: An Exploratory Study,” Journal of Oral Microbiology 10, no. 1 (2018): 1487741, 10.1080/20002297.2018.1487741. 29988721 PMC6032013

[69] G. Rizzatti , L. R. Lopetuso , G. Gibiino , C. Binda , and A. Gasbarrini , “Proteobacteria: A Common Factor in Human Diseases,” BioMed Research International 2017 (2017): 9351507, 10.1155/2017/9351507. 29230419 PMC5688358

[70] L. Zhao , H. Lou , Y. Peng , S. Chen , Y. Zhang , and X. Li , “Comprehensive Relationships Between Gut Microbiome and Faecal Metabolome in Individuals With Type 2 Diabetes and Its Complications,” Endocrine 66, no. 3 (2019): 526–537, 10.1007/s12020-019-02103-8. 31591683

[71] M. Clemente‐Postigo , W. Oliva‐Olivera , L. Coin‐Aragüez , et al., “Metabolic Endotoxemia Promotes Adipose Dysfunction and Inflammation in Human Obesity,” American Journal of Physiology—Endocrinology and Metabolism 316, no. 2 (2019): E319–E332, 10.1152/ajpendo.00277.2018. 30422702

[72] Y. Kashiwagi , S. Aburaya , N. Sugiyama , et al., “ *Porphyromonas gingivalis* Induces Entero‐Hepatic Metabolic Derangements With Alteration of Gut Microbiota in a Type 2 Diabetes Mouse Model,” Scientific Reports 11 (2021): 18398, 10.1038/s41598-021-97868-2. 34526589 PMC8443650

[73] S. E. Pryde , S. H. Duncan , G. L. Hold , C. S. Stewart , and H. J. Flint , “The Microbiology of Butyrate Formation in the Human Colon,” FEMS Microbiology Letters 217, no. 2 (2002): 133–139, 10.1111/j.1574-6968.2002.tb11467.x. 12480096

[74] R. Muñoz‐Tamayo , B. Laroche , E. Walter , et al., “Kinetic Modelling of Lactate Utilization and Butyrate Production by Key Human Colonic Bacterial Species,” FEMS Microbiology Ecology 76, no. 3 (2011): 615–624, 10.1111/j.1574-6941.2011.01085.x. 21388423

[75] A. Simsek , S. Sezer , F. Ozdemir , and M. Haberal , “ *Streptococcus vestibularis* Bacteremia Following Dental Extraction in a Patient on Long‐Term Hemodialysis: A Case Report,” Clinical Kidney Journal 1 (2008): 276–277, 10.1093/ndtplus/sfn071. PMC4421209 25983909

[76] K. L. Tyrrell , D. M. Citron , Y. A. Warren , S. Nachnani , and E. J. C. Goldstein , “Anaerobic Bacteria Cultured From the Tongue Dorsum of Subjects With Oral Malodor,” Anaerobe 9, no. 5 (2003): 243–246, 10.1016/S1075-9964(03)00109-4. 16887710

[77] A. Purohit , B. Kandiyal , S. Kumar , et al., “ *Collinsella aerofaciens* Linked With Increased Ethanol Production and Liver Inflammation Contribute to the Pathophysiology of NAFLD,” iScience 27, no. 2 (2024): 108764, 10.1016/j.isci.2023.108764. 38313048 PMC10837629

[78] L. Lahti , A. Salonen , R. A. Kekkonen , et al., “Associations Between the Human Intestinal Microbiota, *Lactobacillus rhamnosus* GG and Serum Lipids Indicated by Integrated Analysis of High‐Throughput Profiling Data,” PeerJ 1 (2013): e32, 10.7717/peerj.32. 23638368 PMC3628737

[79] S. Astbury , E. Atallah , A. Vijay , G. P. Aithal , J. I. Grove , and A. M. Valdes , “Lower Gut Microbiome Diversity and Higher Abundance of Proinflammatory Genus Collinsella Are Associated With Biopsy‐Proven Nonalcoholic Steatohepatitis,” Gut Microbes 11, no. 3 (2020): 569–580, 10.1080/19490976.2019.1681861. 31696774 PMC7524262

[80] E. Albuquerque‐Souza and S. E. Sahingur , “Periodontitis, Chronic Liver Diseases, and the Emerging Oral‐Gut‐Liver Axis,” Periodontology 2000 89, no. 1 (2022): 125–141, 10.1111/prd.12427. 35244954 PMC9314012

[81] G. Baima , I. Ferrocino , V. Del Lupo , et al., “Effect of Periodontitis and Periodontal Therapy on Oral and Gut Microbiota,” Journal of Dental Research 103, no. 4 (2024): 359–368, 10.1177/00220345231222800. 38362600

[82] S. C. Bischoff , G. Barbara , W. Buurman , et al., “Intestinal Permeability—A New Target for Disease Prevention and Therapy,” BMC Gastroenterology 14 (2014): 189, 10.1186/s12876-014-0189-7. 25407511 PMC4253991

[83] J. Lluch , F. Servant , S. Païssé , et al., “The Characterization of Novel Tissue Microbiota Using an Optimized 16S Metagenomic Sequencing Pipeline,” PLoS One 10, no. 11 (2015): e0142334, 10.1371/journal.pone.0142334. 26544955 PMC4636327

[84] F. F. Anhê , B. A. H. Jensen , T. V. Varin , et al., “Type 2 Diabetes Influences Bacterial Tissue Compartmentalisation in Human Obesity,” Nature Metabolism 2, no. 3 (2020): 233–242, 10.1038/s42255-020-0178-9. 32694777

[85] L. Massier , R. Chakaroun , S. Tabei , et al., “Adipose Tissue Derived Bacteria Are Associated With Inflammation in Obesity and Type 2 Diabetes,” Gut 69, no. 10 (2020): 1796–1806, 10.1136/gutjnl-2019-320118. 32317332

[86] J. Sun , A. Germain , G. Kaglan , et al., “The Visceral Adipose Tissue Bacterial Microbiota Provides a Signature of Obesity Based on Inferred Metagenomic Functions,” International Journal of Obesity 47, no. 10 (2023): 1008–1022, 10.1038/s41366-023-01341-1. 37488221

[87] F. Guérin , “Infections à *Enterobacter cloacae* Complex: Résistance Aux Antibiotiques et Traitement,” Journal des Anti‐Infectieux 17, no. 3 (2015): 79–89, 10.1016/j.antinf.2015.03.002.

[88] X. Lv , L. Zhan , T. Ye , et al., “Gut Commensal *Agathobacter rectalis* Alleviates Microglia‐Mediated Neuroinflammation Against Pathogenesis of Alzheimer Disease,” iScience 27, no. 11 (2024): 111116, 10.1016/j.isci.2024.111116. 39498309 PMC11532950

[89] J. J. Maki , M. Howard , S. Connelly , M. A. Pettengill , D. J. Hardy , and A. Cameron , “Species Delineation and Comparative Genomics Within the *Campylobacter ureolyticus* Complex,” Journal of Clinical Microbiology 61, no. 5 (2023): e00046‐23, 10.1128/jcm.00046-23. 37129508 PMC10204631

[90] E. R. K. Eribe , B. J. Paster , D. A. Caugant , et al., “Genetic Diversity of Leptotrichia and Description of *Leptotrichia goodfellowii* sp. nov., *Leptotrichia hofstadii* sp. nov., *Leptotrichia shahii* sp. nov. and *Leptotrichia wadei* sp. nov,” International Journal of Systematic and Evolutionary Microbiology 54, no. 2 (2004): 583–592, 10.1099/ijs.0.02819-0. 15023979

[91] R. P. Ellen , “Establishment and Distribution of Actinomyces Viscosus and *Actinomyces naeslundii* in the Human Oral Cavity,” Infection and Immunity 14, no. 5 (1976): 1119–1124, 10.1128/iai.14.5.1119-1124.1976. 977124 PMC415502

[92] Y. C. Hsiao , Y. H. Lee , C. M. Ho , C. H. Tseng , and J. H. Wang , “Clinical Characteristics of *Actinomyces viscosus* Bacteremia,” Medicina (Kaunas, Lithuania) 57, no. 10 (2021): 10, 10.3390/medicina57101064. PMC8537041 34684101

[93] K. Diop , D. Raoult , F. Bretelle , and F. Fenollar , ““*Ezakiella massiliensis*” sp. nov., a New Bacterial Species Isolated From Human Female Genital Tract,” New Microbes and New Infections 15 (2016): 16, 10.1016/j.nmni.2016.09.011. 27843546 PMC5099271

[94] B. G. Loos , J. Craandijk , F. J. Hoek , P. M. Wertheim‐van Dillen , and U. van der Velden , “Elevation of Systemic Markers Related to Cardiovascular Diseases in the Peripheral Blood of Periodontitis Patients,” Journal of Periodontology 71, no. 10 (2000): 1528–1534, 10.1902/jop.2000.71.10.1528. 11063384

[95] B. Noack , R. J. Genco , M. Trevisan , S. Grossi , J. J. Zambon , and E. De Nardin , “Periodontal Infections Contribute to Elevated Systemic C‐Reactive Protein Level,” Journal of Periodontology 72, no. 9 (2001): 1221–1227, 10.1902/jop.2000.72.9.1221. 11577954

[96] T. Bonsmann , M. Mochol , E. Bonsmann , et al., “The Role of IL‐17 in Periodontitis and Its Systemic Connections,” International Journal of Molecular Sciences 26, no. 22 (2025): 10902, 10.3390/ijms262210902. 41303386 PMC12652818

[97] S. Lê , E. Sturaro , C. Thomas , et al., “ *Porphyromonas gingivalis* ‐Induced Glucose Intolerance During Periapical Lesions Requires Its LPS Throught a Th17 Immune Response,” International Journal of Oral Science 17, no. 1 (2025): 69, 10.1038/s41368-025-00403-6. 41233340 PMC12615820

[98] V. Blasco‐Baque , L. Garidou , C. Pomié , et al., “Periodontitis Induced by *Porphyromonas gingivalis* Drives Periodontal Microbiota Dysbiosis and Insulin Resistance via an Impaired Adaptive Immune Response,” Gut 66, no. 5 (2017): 872–885, 10.1136/gutjnl-2015-309897. 26838600 PMC5531227

[99] S. Yoshida , M. Hatasa , Y. Ohsugi , et al., “ *Porphyromonas gingivalis* Administration Induces Gestational Obesity, Alters Gene Expression in the Liver and Brown Adipose Tissue in Pregnant Mice, and Causes Underweight in Fetuses,” Frontiers in Cellular and Infection Microbiology 11 (2022): 745117, 10.3389/fcimb.2021.745117. 35096633 PMC8792863

[100] M. O. Li , Y. Y. Wan , S. Sanjabi , A. K. L. Robertson , and R. A. Flavell , “Transforming Growth Factor‐Beta Regulation of Immune Responses,” Annual Review of Immunology 24 (2006): 99–146, 10.1146/annurev.immunol.24.021605.090737. 16551245

[101] T. A. Wynn , “Cellular and Molecular Mechanisms of Fibrosis,” Journal of Pathology 214, no. 2 (2008): 199–210, 10.1002/path.2277. 18161745 PMC2693329

[102] P. Dahiya , R. Kamal , R. Gupta , and H. Saini , “Evaluation of the Serum Antioxidant Status in Patients With Chronic Periodontitis,” Indian Journal of Multidisciplinary Dentistry 6, no. 1 (2016): 3, 10.4103/2229-6360.188213.

[103] B. Halliwell , “Reactive Oxygen Species in Living Systems: Source, Biochemistry, and Role in Human Disease,” American Journal of Medicine 91, no. 3C (1991): 14S–22S, 10.1016/0002-9343(91)90279-7. 1928205

[104] M. I. Fredriksson , A. K. Gustafsson , K. G. Bergström , and B. E. Asman , “Constitutionally Hyperreactive Neutrophils in Periodontitis,” Journal of Periodontology 74, no. 2 (2003): 219–224, 10.1902/jop.2003.74.2.219. 12666711

[105] S. Furukawa , T. Fujita , M. Shimabukuro , et al., “Increased Oxidative Stress in Obesity and Its Impact on Metabolic Syndrome,” Journal of Clinical Investigation 114, no. 12 (2004): 1752–1761, 10.1172/JCI21625. 15599400 PMC535065

[106] A. Baker , C. C. Lin , C. Lett , B. Karpinska , M. H. Wright , and C. H. Foyer , “Catalase: A Critical Node in the Regulation of Cell Fate,” Free Radical Biology & Medicine 199 (2023): 56–66, 10.1016/j.freeradbiomed.2023.02.009. 36775107

[107] Y. Wang , O. Andrukhov , and X. Rausch‐Fan , “Oxidative Stress and Antioxidant System in Periodontitis,” Frontiers in Physiology 8 (2017): 910, 10.3389/fphys.2017.00910. 29180965 PMC5693842

[108] K. Panjamurthy , S. Manoharan , and C. R. Ramachandran , “Lipid Peroxidation and Antioxidant Status in Patients With Periodontitis,” Cellular & Molecular Biology Letters 10, no. 2 (2005): 255–264. 16010291

[109] N. Houstis , E. D. Rosen , and E. S. Lander , “Reactive Oxygen Species Have a Causal Role in Multiple Forms of Insulin Resistance,” Nature 440, no. 7086 (2006): 944–948, 10.1038/nature04634. 16612386

[110] E. M. Allen , J. B. Matthews , D. J. O' Halloran , H. R. Griffiths , and I. L. Chapple , “Oxidative and Inflammatory Status in Type 2 Diabetes Patients With Periodontitis: Periodontitis and Diabetes Inflammatory Status,” Journal of Clinical Periodontology 38, no. 10 (2011): 894–901, 10.1111/j.1600-051X.2011.01764.x. 21883360

[111] P. White , P. Cooper , M. Milward , and I. Chapple , “P95—Differential Activation of Neutrophil Extracellular Traps by Specific Periodontal Bacteria,” Free Radical Biology and Medicine 75 (2014): S53, 10.1016/j.freeradbiomed.2014.10.827. 26461408

[112] J. Katz , M. Y. Flugelman , A. Goldberg , and M. Heft , “Association Between Periodontal Pockets and Elevated Cholesterol and Low Density Lipoprotein Cholesterol Levels,” Journal of Periodontology 73, no. 5 (2002): 494–500, 10.1902/jop.2002.73.5.494. 12027250

[113] P. Basdorf , T. Kocher , S. E. Baumeister , et al., “Periodontitis Adversely Affects Lipoprotein Subfractions—Results From the Cohort Study SHIP‐TREND: Periodontitis Adversely Affects Lipoprotein Subfractions,” Diabetes & Metabolism 50, no. 6 (2024): 101584, 10.1016/j.diabet.2024.101584. 39396553

[114] H. Miyazawa , K. Tabeta , S. Miyauchi , et al., “Effect of *Porphyromonas gingivalis* Infection on Post‐Transcriptional Regulation of the Low‐Density Lipoprotein Receptor in Mice,” Lipids in Health and Disease 11 (2012): 121, 10.1186/1476-511X-11-121. 22992388 PMC3503659

[115] T. Maekawa , N. Takahashi , K. Tabeta , et al., “Chronic Oral Infection With *Porphyromonas gingivalis* Accelerates Atheroma Formation by Shifting the Lipid Profile,” PLoS One 6, no. 5 (2011): e20240, 10.1371/journal.pone.0020240. 21625524 PMC3098290

[116] F. D'Aiuto , M. Parkar , G. Andreou , et al., “Periodontitis and Systemic Inflammation: Control of the Local Infection Is Associated With a Reduction in Serum Inflammatory Markers,” Journal of Dental Research 83, no. 2 (2004): 156–160, 10.1177/154405910408300214. 14742655

[117] W. J. Teeuw , D. E. Slot , H. Susanto , et al., “Treatment of Periodontitis Improves the Atherosclerotic Profile: A Systematic Review and Meta‐Analysis,” Journal of Clinical Periodontology 41, no. 1 (2014): 70–79, 10.1111/jcpe.12171. 24111886

[118] Y. W. Fu , X. X. Li , H. Z. Xu , Y. Q. Gong , and Y. Yang , “Effects of Periodontal Therapy on Serum Lipid Profile and Proinflammatory Cytokines in Patients With Hyperlipidemia: A Randomized Controlled Trial,” Clinical Oral Investigations 20 (2016): 1263–1269, 10.1007/s00784-015-1621-2. 26434651

[119] G. Utermann , C. Haibach , M. Trommsdorff , et al., “Genetic Architecture of the Atherogenic Lipoprotein(A),” Annals of the New York Academy of Sciences 748 (1995): 301–311; discussion 311–312, 10.1111/j.1749-6632.1994.tb17328.x. 7695174

[120] D. Rhainds , M. R. Brodeur , and J. C. Tardif , “Lipids, Apolipoproteins, and Inflammatory Biomarkers of Cardiovascular Risk: What Have We Learned?,” Clinical Pharmacology and Therapeutics 104, no. 2 (2018): 244–256, 10.1002/cpt.1114. 29761474

[121] M. Sanz , A. Marco del Castillo , S. Jepsen , et al., “Periodontitis and Cardiovascular Diseases: Consensus Report,” Journal of Clinical Periodontology 47, no. 3 (2020): 268–288, 10.1111/jcpe.13189. 32011025 PMC7027895

